# Usability of reference-free transcriptome assemblies for detection of differential expression: a case study on *Aethionema arabicum* dimorphic seeds

**DOI:** 10.1186/s12864-019-5452-4

**Published:** 2019-01-30

**Authors:** Per K. I. Wilhelmsson, Jake O. Chandler, Noe Fernandez-Pozo, Kai Graeber, Kristian K. Ullrich, Waheed Arshad, Safina Khan, Johannes A. Hofberger, Karl Buchta, Patrick P. Edger, J. Chris Pires, M. Eric Schranz, Gerhard Leubner-Metzger, Stefan A. Rensing

**Affiliations:** 1 Plant Cell Biology, Faculty of Biology, University of Marburg, 35043 Marburg, Germany; 2 School of Biological Sciences, Royal Holloway University of London, Egham, Surrey TW20 0EX UK; 3 Biosystematics Group, Wageningen University, Wageningen, 6708 PB The Netherlands; 4 Department of Horticulture, Michigan State University, East Lansing, MI 48864 USA; 5 Division of Biological Sciences, University of Missouri, Columbia, MO 65211 USA; 6 Laboratory of Growth Regulators, Centre of the Region Haná for Biotechnological and Agricultural Research, Palacký University and Institute of Experimental Botany, Academy of Sciences of the Czech Republic, 78371 Olomouc, Czech Republic; 7 BIOSS Centre for Biological Signalling Studies, University of Freiburg, Freiburg, Germany; 8 Present Address: Max Planck Institute for Evolutionary Biology, August-Thienemann-Straße 2, 24306 Ploen, Germany

**Keywords:** *Aethionema arabicum*, Dimorphic seeds, Reference and reference-free, RNA-seq, Transcriptome

## Abstract

**Background:**

RNA-sequencing analysis is increasingly utilized to study gene expression in non-model organisms without sequenced genomes. *Aethionema arabicum* (Brassicaceae) exhibits seed dimorphism as a bet-hedging strategy – producing both a less dormant mucilaginous (M^+^) seed morph and a more dormant non-mucilaginous (NM) seed morph. Here, we compared de novo and reference-genome based transcriptome assemblies to investigate *Ae. arabicum* seed dimorphism and to evaluate the reference-free versus -dependent approach for identifying differentially expressed genes (DEGs).

**Results:**

A de novo transcriptome assembly was generated using sequences from M^+^ and NM *Ae. arabicum* dry seed morphs. The transcripts of the de novo assembly contained 63.1% complete Benchmarking Universal Single-Copy Orthologs (BUSCO) compared to 90.9% for the transcripts of the reference genome. DEG detection used the strict consensus of three methods (DESeq2, edgeR and NOISeq). Only 37% of 1533 differentially expressed de novo assembled transcripts paired with 1876 genome-derived DEGs. Gene Ontology (GO) terms distinguished the seed morphs: the terms translation and nucleosome assembly were overrepresented in DEGs higher in abundance in M^+^ dry seeds, whereas terms related to mRNA processing and transcription were overrepresented in DEGs higher in abundance in NM dry seeds. DEGs amongst these GO terms included ribosomal proteins and histones (higher in M^+^), RNA polymerase II subunits and related transcription and elongation factors (higher in NM). Expression of the inferred DEGs and other genes associated with seed maturation (e.g. those encoding late embryogenesis abundant proteins and transcription factors regulating seed development and maturation such as ABI3, FUS3, LEC1 and WRI1 homologs) were put in context with *Arabidopsis thaliana* seed maturation and indicated that M^+^ seeds may desiccate and mature faster than NM. The 1901 transcriptomic DEG set GO-terms had almost 90% overlap with the 2191 genome-derived DEG GO-terms.

**Conclusions:**

Whilst there was only modest overlap of DEGs identified in reference-free versus -dependent approaches, the resulting GO analysis was concordant in both approaches. The identified differences in dry seed transcriptomes suggest mechanisms underpinning previously identified contrasts between morphology and germination behaviour of M^+^ and NM seeds.

**Electronic supplementary material:**

The online version of this article (10.1186/s12864-019-5452-4) contains supplementary material, which is available to authorized users.

## Background

RNA-sequencing (RNA-seq) technology is a valuable tool to investigate gene expression [1], especially in species where no reference genome is available. Without any prior molecular data about a particular species, de novo transcriptome assembly of RNA-seq data offers a unique opportunity to study gene expression on a transcriptome-wide scale of any trait of interest. Due to drops in library and sequencing costs, it is now widely utilized by many scientists to study traits of particular interest in a wide-range of species. However, there are limitations to using a de novo transcriptome assembly compared to a reference-genome guided approach. Since less sequence information is used in the creation of the transcripts in a de novo transcriptome, in comparison to a reference genome, low expressed genes are more difficult to detect. De novo assembled transcripts are also more likely to be fragmented.

Here, we apply a reference-free and a reference-dependent approach to compare the gene expression in the dry mature dimorphic seeds of *Aethionema arabicum*. This species represents the sister lineage to all other Brassicaceae, and is a herbaceous annual native to parts of Eastern Europe and the Middle East. It exhibits diaspore heteromorphism – i.e. the ability to produce multiple morphologically and physiologically distinct fruit or seed morphs on individual plants [2, 3]. *Ae. arabicum* produces two distinct fruits, a dehiscent (DEH) and an indehiscent (IND) fruit morph. The dehiscent fruit contains typically four seeds, shatters on maturity, and disperses mucilaginous seeds (M^+^). Conversely, the indehiscent fruit contains a single non-mucilaginous seed (M^−^) encased in a pericarp (fruit coat). Upon maturity, the entire IND fruit detaches, via abscission, from the parent plant leading to the fruit’s dispersal [3, 4]. In addition to these morphological differences between the two morphs, the NM seeds appear to be more dormant compared to the M^+^ seeds, with NM exhibiting much slower germination at 14°C [3]. The production of two contrasting seed/fruit morphs is proposed to constitute a bet-hedging strategy that increases long-term plant fitness in disturbed and unpredictable extreme environments. However, how this heteromorphism is reflected at the transcriptomic level is unknown. With its recently published genome sequence and its basal phylogenetic position within the Brassicaceae, *Ae. arabicum* has potential as a model species for diaspore heteromorphism [3, 5].

For many other non-model plant species, including other heteromorphic systems, a reference genome is not available. Thus, comparing the effectiveness of reference-free and reference-dependent transcriptome analyses is pertinent to future investigations into such non-model species. Comparison of the transcriptomes of the two *Ae. arabicum* seed morphs represents a realistic and interesting demonstration of both approaches. There are many genomes with accompanying large sets of microarray and qRT-PCR data, and it was early on concluded that de novo assembled transcriptome expression profiles positively correlate with corresponding microarrays and qRT-PCRs [6–8]. Due to the potential of RNA-seq, much work has been done on how to get the best results out of a de novo transcriptome assembly [9–13]. The Trinity suite [14] is one of the most cited de novo transcriptome assemblers exhibiting good performance metrics [13]. In order to generate a representative transcriptome, sequencing depth is important to be able to reconstruct as many genes as possible including those expressed at low levels. The ability to detect weakly expressed sequences can only be improved by increasing the sequencing depth. This highlights the diminishing investment returns (sequencing depth) in relation to yield (sequence resolution) for RNA-seq. Despite the known limiting factors of transcriptome assembly, the knowledge gained per investment makes reference-free gene expression profiling an obvious choice when working with non-model species.

To evaluate the knowledge that can be gained with reference-free gene expression profiling, a reference-dependent expression profiling was carried out using the existing genome assembly of *Ae. arabicum* [5]. To investigate the seed dimorphism of *Ae. arabicum*, we conducted a highly robust differentially expressed genes (DEGs) detection analysis and used it to compare DEGs derived from a transcriptome-based and a genome-based mapping approach. The aim of this study was to find DEGs between *Ae. arabicum* dimorphic seeds, and to compare the RNA-seq analysis performed using two different references, a de novo transcriptome assembly and the *Ae. arabicum* genome sequence V2.5.

## Results and discussion

### Overview of RNA-seq analysis of *Ae. arabicum* mature dimorphic seeds

**Fig. 1:**
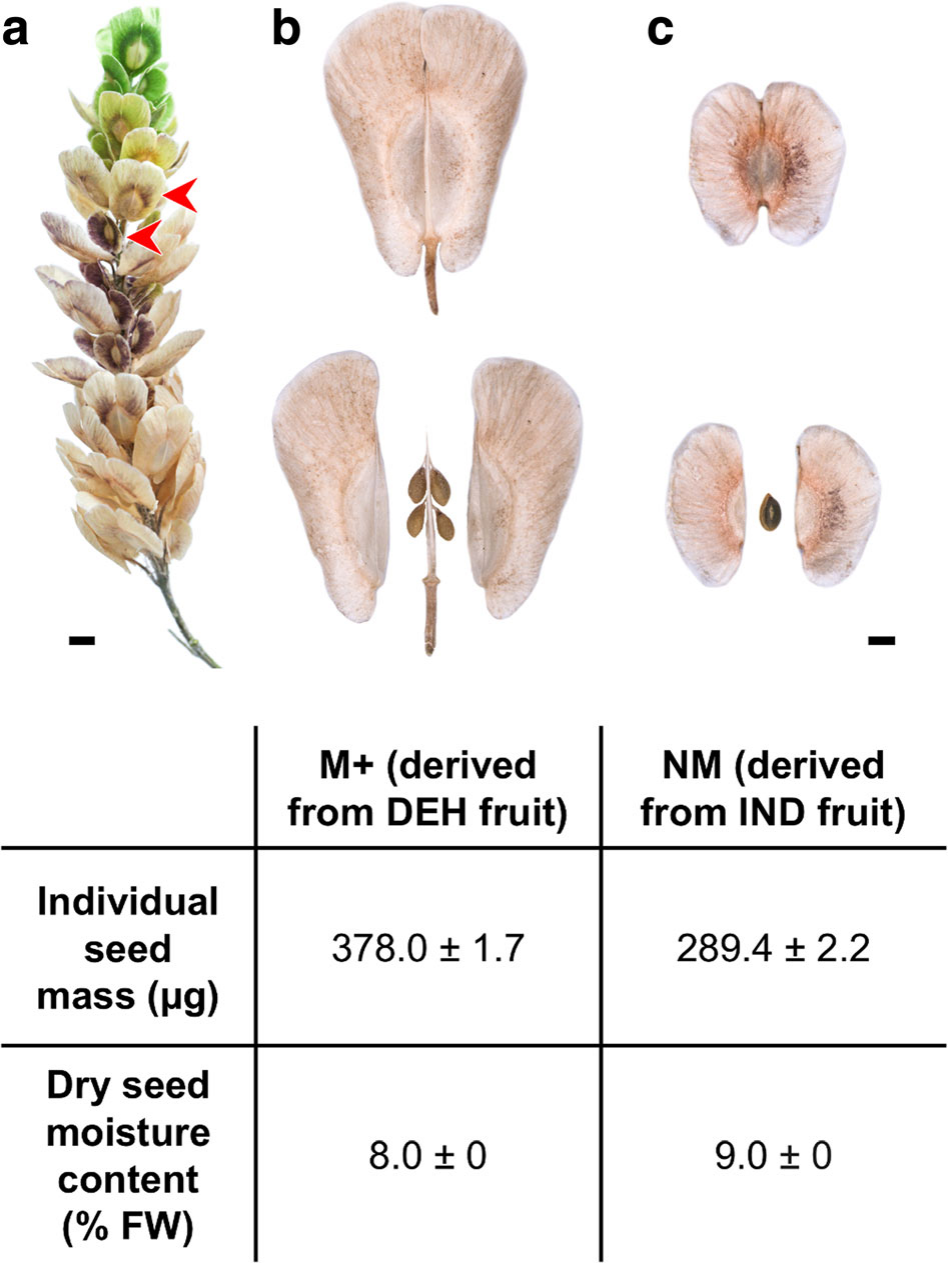
Fruit and seed dimorphism in *Ae. arabicum.* Mature infructescence (**a**) of *Ae. arabicum*, showing distinct dehiscent (DEH) and indehiscent (IND) fruit morphs (marked by red arrows). Large DEH fruits (**b**) contain up to six mucilaginous (M^+^) seeds, while small IND fruits (**c**) contain a single, non-mucilaginous (NM) seed. Both seed morphs differ in mean seed mass and moisture content. Values shown are means ± SEM for *n* = 8 each of 100 seeds (mass), and *n* = 4 each of 30 seeds (moisture content) replicate measurements. Scale bars = 4 mm (**a**), 1 mm (**b** and **c**). FW, fresh weight

**Fig. 2:**
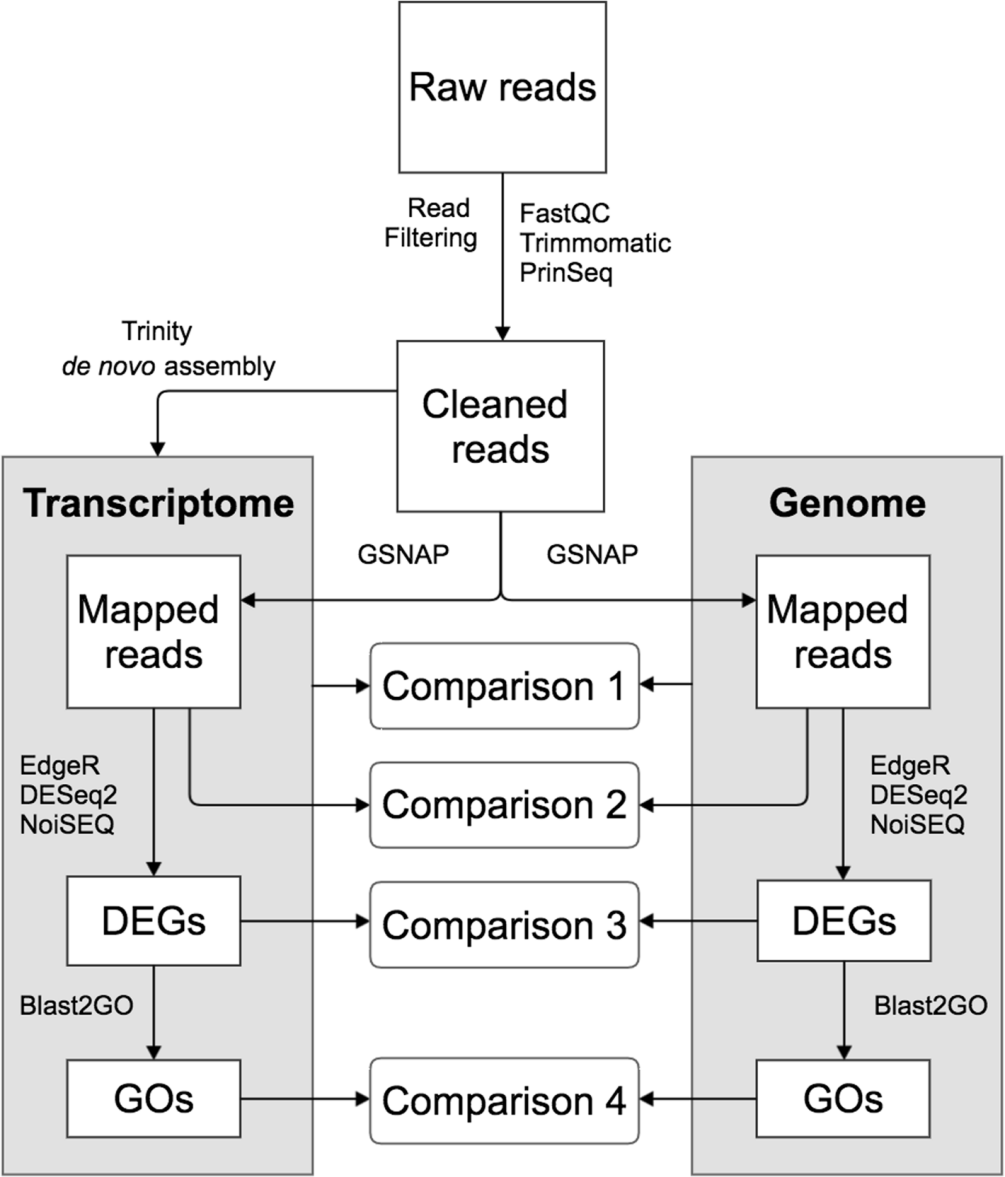
RNA-Seq analysis pipeline. Raw RNA-seq reads were checked for quality control (FastQC) and processed to remove adapters and low-quality bases (Trimmomatic, PrinSeq). Cleaned reads were either: mapped to the genome (GSNAP); or were used for de novo *transcriptome* assembly (Trinity) and mapped to the resulting transcriptome (GSNAP). Transcriptome-mapped and genome-mapped reads were compared at each stage of analysis: After mapping; after differentially expressed gene (DEG) identification (EdgeR, DESeq2, NoiSEQ), and after gene-ontology (GO) analysis (Blast2GO)

The mature dimorphic seeds, M^+^ from DEH fruits and NM from IND fruits (designated NM, for “non-mucilaginous”, in our RNA-seq analysis), differed in size and mass but not in seed moisture content (Fig. 1). RNA was extracted from freshly harvested mature M^+^ and NM seeds and the resultant RNA samples processed as described in the Methods section. As shown in Fig. 2, RNA-seq raw reads were processed and checked using FastQC (https://www.bioinformatics.babraham.ac.uk/projects/fastqc/), Trimmomatic version 0.32 [15] and PrinSeq [16]. Subsequently, cleaned reads were used for de novo transcriptome assembly for *Ae. arabicum* M^+^ and NM seeds using Trinity [14]. The same set of cleaned reads was mapped to the gene models of the reference genome using GSNAP [17]. EdgeR, DESeq2 and NOISeq [18–20] were used to normalize read counts and to detect DEGs in a strict consensus approach, and Blast2GO [21] was used to assign Gene Ontology (GO) terms to the genes. Comparisons were performed between the transcriptome and the genome (Comparison 1, Fig. 2), the reads mapped to both the de novo transcriptome and reference-based genes (Comparison 2, Fig. 2), the DEGs found in both approaches (Comparison 3, Fig. 2), and between their GO terms (Comparison 4, Fig. 2).

### Read filtering of RNA-seq raw data

**Fig. 3:**
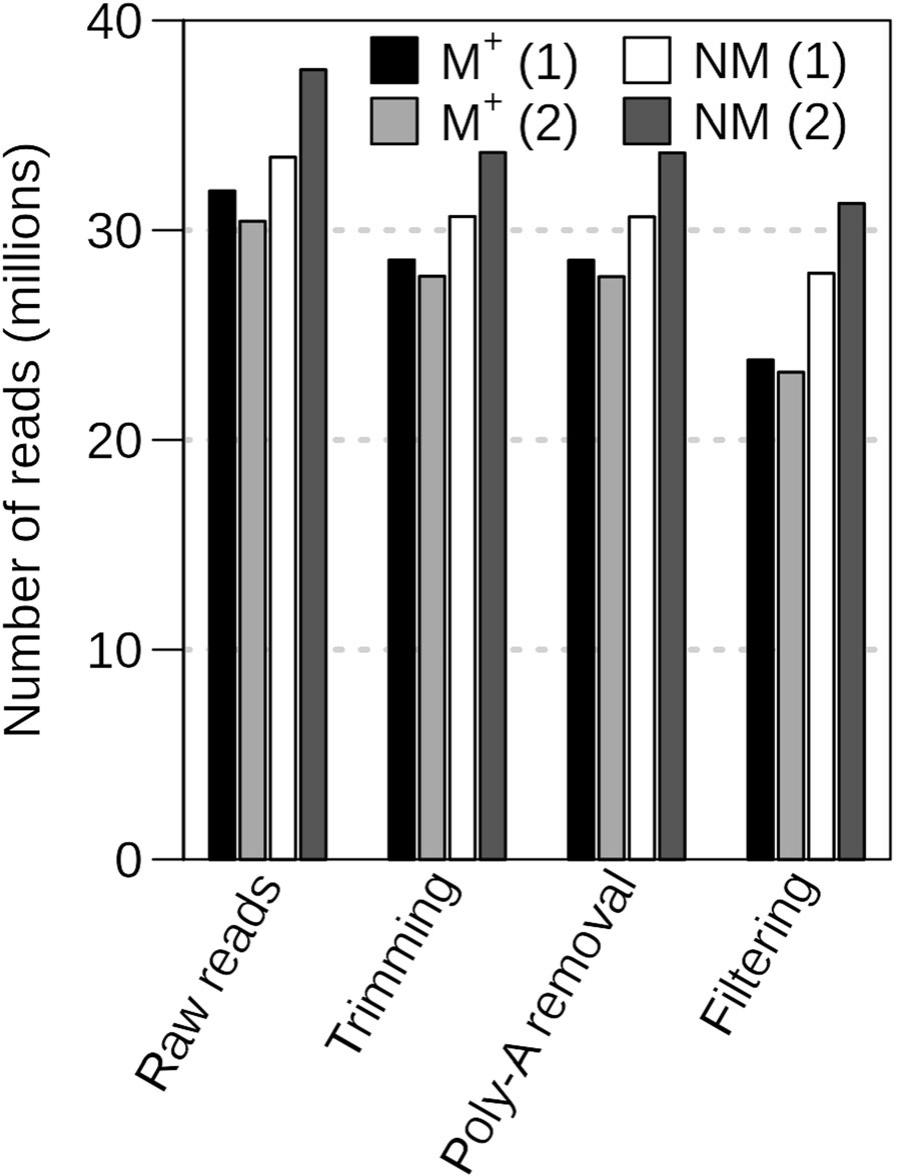
From raw to filtered reads. Trimming of raw reads with Trimmomatic removed adapters and low-quality reads. Trimmed reads were further processed with poly-A / poly-T removal with PrinSeq. The resulting reads were then filtered to remove chloroplastic, mitochondrial and ribosomal RNA reads. The total number of reads left after each step is indicated for samples M^+^ (1), M^+^ (2), NM (1) and NM (2)

To generate the raw reads, a total of four cDNA libraries were sequenced, with two biological replicates of *Ae. arabicum* dry mature dimorphic seeds, termed M^+^ 1/M^+^ 2 for the M^+^ seeds and NM1/NM2 for the NM seeds. Raw reads were processed to remove adapters, organellar, ribosomal RNA (rRNA) and low-quality sequences (Fig. 3). Adapter sequences were removed and low-quality sequences were trimmed using Trimmomatic. Poly-A and poly-T tails were removed using PrinSeq. This process resulted in an average loss of 9.6% of all reads for the four libraries. To reduce the complexity of the assembly/mapping, and to check for correct poly-T selection, all data were filtered to remove reads with plastid, mitochondrial and ribosomal RNA origin resulting in an average loss of 12% of the reads for the four libraries. Visualization of these quality control steps provides a good measure of library quality making possible to see if there are any higher than average read losses in the individual steps. After passing all the filters, the sets of cleaned sequences contained between 20 and 30 million reads (Fig. 3), which is in the range of read numbers commonly used for RNA-seq analysis for DEG detection [22].

### De novo transcriptome assembly

**Fig. 4:**
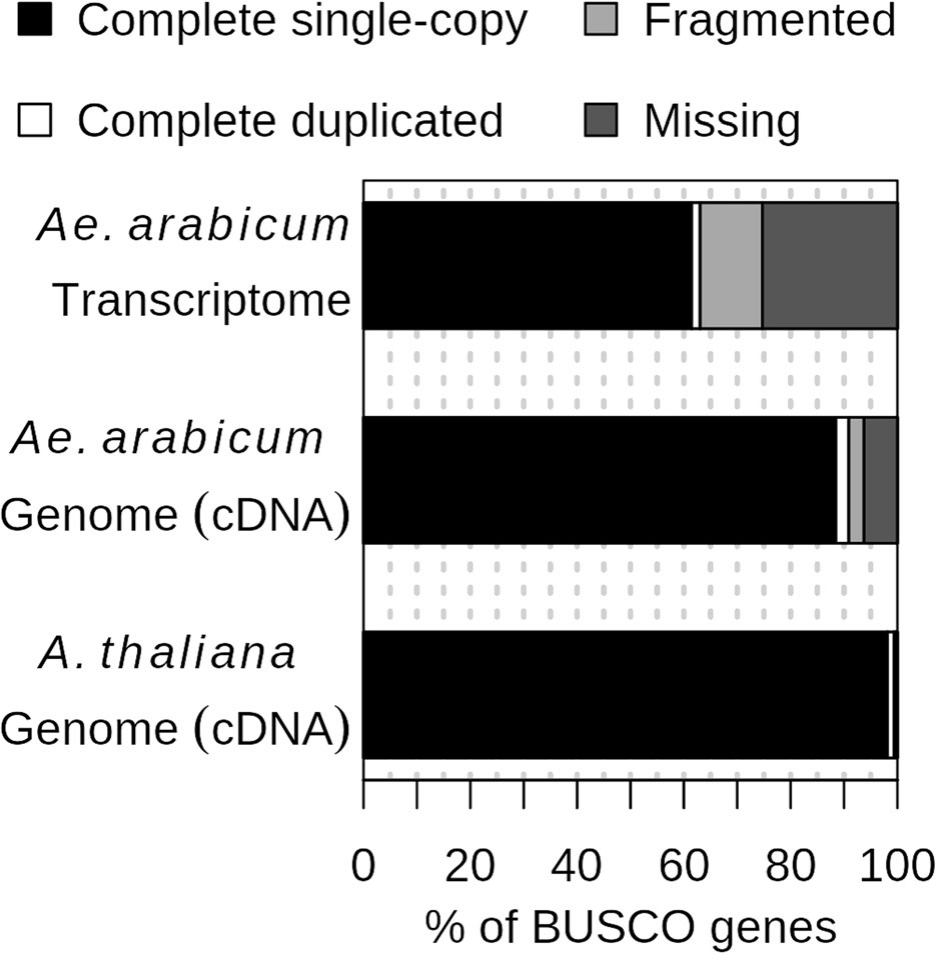
BUSCO completeness analysis. cDNA from the *Ae. arabicum* de novo assembly, *Ae. arabicum* genome v2.5 and *A. thaliana* TAIR10 were compared to 1440 Embryophyta reference orthologs for completeness assessment

**Fig. 5:**
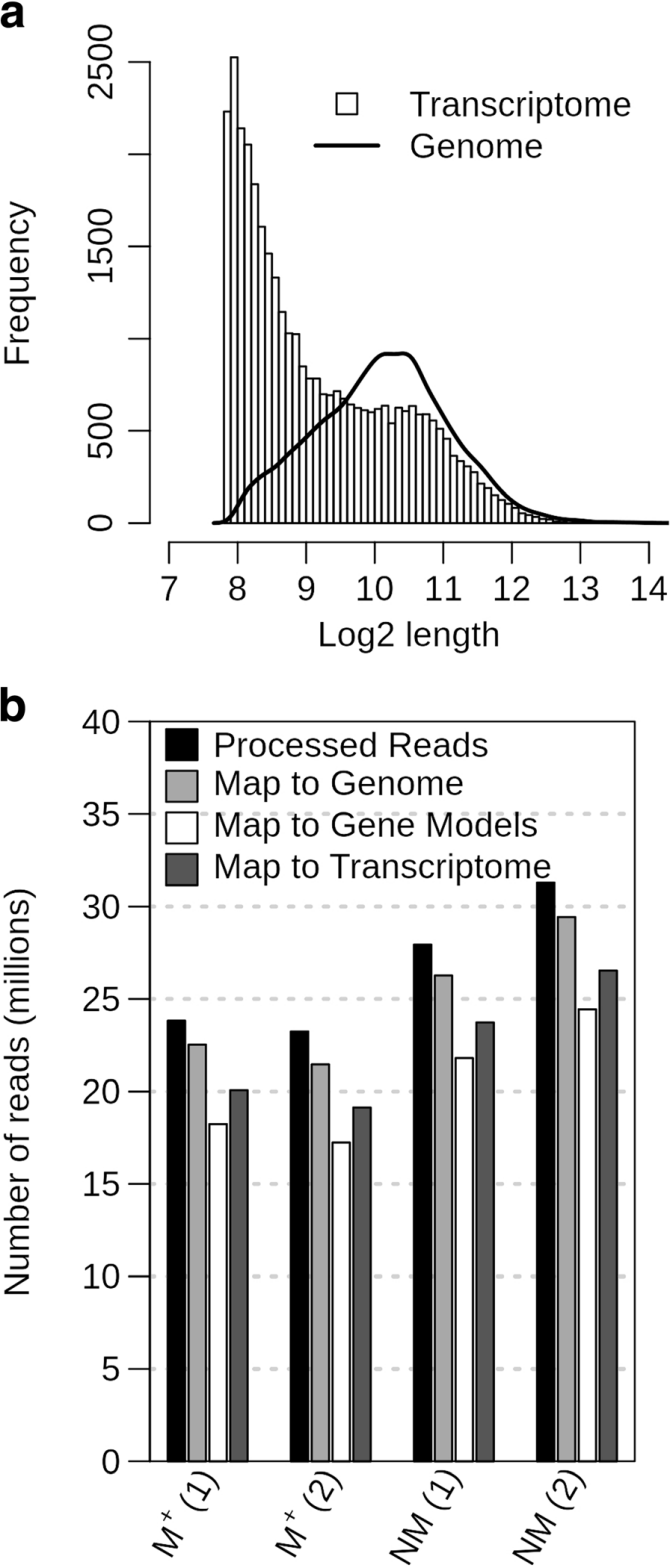
Transcript length distribution and mapping efficiency. Length distribution of the de novo assembled transcripts and *Ae. arabicum* mRNAs derived from the genome assembly (**a**)**.** Processed reads and amount of mapping reads to the *Ae. arabicum* whole genome V2.5, gene models from V2.5**,** and de novo assembly transcripts (**b**)

Processed reads from all four samples combined were assembled de novo using Trinity to reconstruct the *Ae. arabicum* dry seed transcriptome. From a total of 30,742,186 reads, 27,407,363 reads (89.15%) could be assembled. This resulted in a total of 62,182 transcripts including potential splice variants or fragmentary sequences. The longest gene sequences from each Trinity gene cluster were selected to reduce redundancy, resulting in 34,784 transcripts (Additional file 1). To assess the quality and completeness of the *Ae. arabicum* dry seed de novo transcriptome, and to compare it to the gene models from the genome (Comparison 1, Fig. 2), it was analyzed using the Benchmarking Universal Single-Copy Orthologs (BUSCO) tool [23] (embryophyta *odb9*) which checks for the presence of Embryophyta “near-universal single-copy orthologs”. For the de novo assembled transcriptome, 908 transcripts out of 1440 of the BUSCO genes were complete (63.1%). Of those, 885 were single copy and 23 duplicated. One hundred sixty-eight transcripts were fragmented and 364 missing (Fig. 4). The corresponding number of BUSCO completeness in the 23,594 gene models of the genome was 1309 (90.9%). Of those, 1274 were single copy and 35 duplicated. Forty-one gene models were fragmented and 90 missing (Fig. 4). To compare these results with a well-annotated model species, *Arabidopsis thaliana* (TAIR10, [24]) was included in the BUSCO analysis. For *A. thaliana*, 1431 complete genes were found (99.3%), 1413 were single copy and 18 duplicated; five genes were fragmented and four missing. The relatively low number of complete genes in *Ae. arabicum* transcriptome is to be expected, since dry seeds represent an atypical tissue that lacks much of the transcription going on in photosynthetically/developmentally active tissue. Also, it is common that some genes are fragmented in de novo assemblies, as shown in Fig. 5a which indicates the length distribution of de novo assembled transcripts is skewed towards shorter lengths compared to the *Ae. arabicum* mRNAs predicted from the genome.

### Mapping reads to the transcriptome and the genome

To determine read counts for subsequent DEG analysis, cleaned reads were mapped to the transcriptome and the genome using GSNAP [17] and counted using HTSeq-count [25] with the respective general feature format (GFF) file. Counted reads for the four samples are shown in Fig. 5b. This analysis showed that on average 84% of reads were mapped to the transcriptome and 94% to the genome. The drop from 89.15% of the reads being used for assembling to 84% mapping is to a large extent explained by the removal of redundancy keeping only the longest isoform of each transcript. On average, the cleaned reads had a read length of 83 bp. Mapping the reads to the 23,594 genomic gene models, 7814 models had a coverage lower than 1 (where 1 corresponds to an average 1-fold coverage of the gene length; see Methods for details) and 11,189 gene models had a coverage lower than 5 (Additional file 2: Table S1). This highlights the challenges to assemble full-length transcripts. Using reciprocal BLASTN with a coverage cut off of 50% for both transcriptomic (virtual transcripts) and genomic coding sequences (CDS), 6745 transcript-gene pairs could be identified (Additional file 2: Table S2). To compare the expression levels between the transcriptome- and the genome-based approach (Comparison 2, Fig. 2), the 6745 gene-transcript pairs were considered. Principal Component Analysis (PCA) using the Reads Per Kilobase per Million mapped reads (RPKM) of the 6745 genes (Additional file 3: Figure S1) showed, as expected [9], that replicates from the same seed morph clustered together and samples from different seed morphs are more distant. This is apparent in both the de novo and reference-genome approach. To assess gene family completeness, the predicted proteins of the reference genome and the de novo transcriptome were screened for Transcription Associated Proteins (TAPs, comprising transcription factors, TF, and transcriptional regulators, TR) using the TAPscan pipeline [26]. 1860 (113 unique families) and 1009 (105 unique families) TAPs were detected in the genome and transcriptome, respectively (Additional file 2: Table S3 and S4). Finding fewer TAPs in the transcriptome is to be expected due to the atypical tissue of the transcriptome in comparison to the whole genome. Genome-wide, 7.6% were multi domain TAPs (defined by more than one domain), while only 4.2% TAPs were multi domain in the transcriptome, due to the fragmented nature of the transcriptome.

### Differential gene expression analysis

**Fig. 6:**
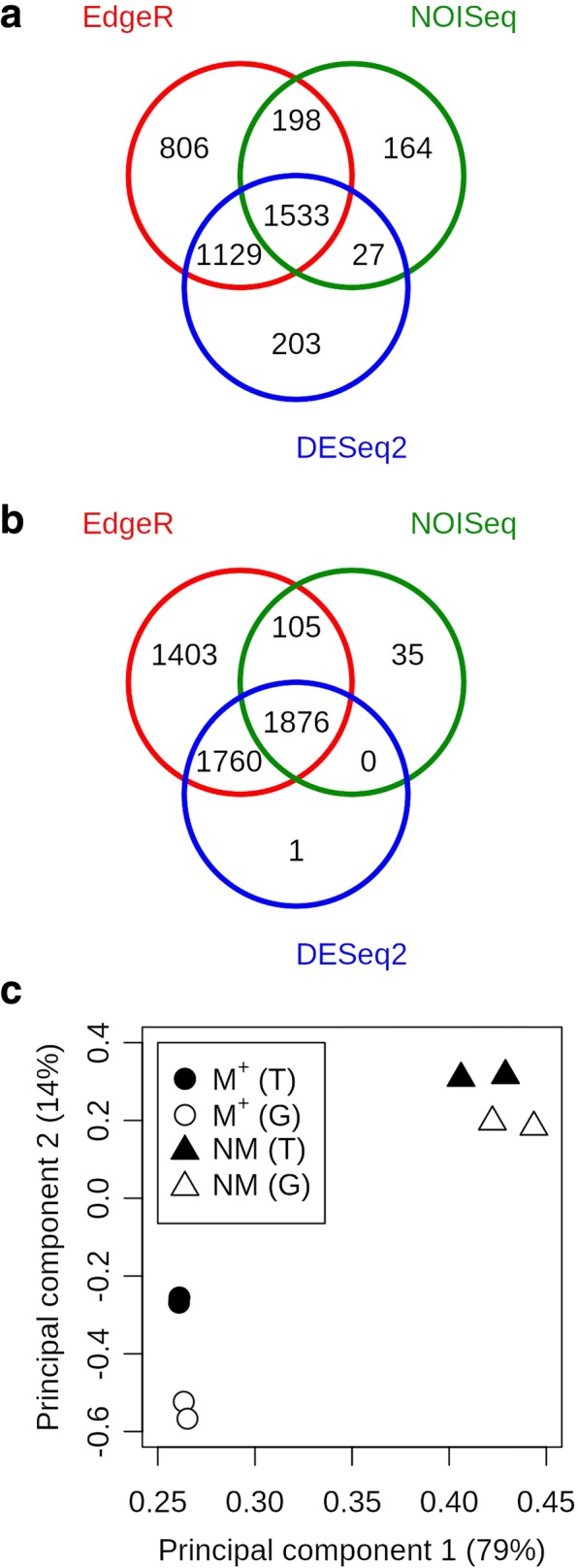
Consensus of DEG calling and PCA of overlap of common DEGs. Venn diagram of the DEGs called between NM and M^+^ seeds by the three DEG detection programs (edgeR, NOIseq and DESeq2) using the transcriptome (**a**) and genome (**b**) approach. Principal Component Analysis of RPKM (Reads Per Kilobase per Million reads) of the 561 DEGs common to the transcriptome, ‘T’ and genome, ‘G’ (**c**). Samples M^+^ (circle) and NM (triangles), in black, show the results for the dehiscent and indehiscent seeds in the transcriptome approach. Samples M^+^ (circle) and NM (triangles), in white, show the corresponding results in the genome approach. The percentage variance explained by each principal component is indicated on the axes

To learn more about the differences between the mature dimorphic seeds, gene expression was analyzed using both references: the de novo transcriptome assembly and the genome annotation. Since the combination of several methods minimizes false positives [27], DEGs were detected in a robust way using the strict consensus (overlap) of three different DEG analysis programs: edgeR, DESeq2 and NOISeq. This approach combines two parametric methods to detect DEGs (edgeR and DESeq2), and a non-parametric method (NOISeq). The intersection of the DEGs obtained by the three methods was considered the resulting DEGs (Fig. 6a, b). In all comparisons edgeR called the most DEGs while NOISeq was the most restrictive (Fig. 6a, b), thus the NOISeq set was representing the consensus DEG set best. This approach resulted in the exclusion of low expressed DEGs (Additional file 3: Figure S2) below RPKM 2, representing genes of low abundance that typically cannot be shown as expressed in a quantitative PCR approach [28].

One thousand five hundred thirty-three and one thousand eight hundred seventy-six DEGs were obtained, respectively, using the de novo transcriptome (Fig. 6a, Additional file 2: Table S4) and the reference genome (Fig. 6b; Additional file 2: Table S3). When comparing common DEGs detected in both approaches (Comparison 3, Fig. 2), 561 gene-transcript pairs were found to be differentially expressed in both. Thus, 561/1533 (37%) of the de novo transcriptome consensus DEGs were also well represented by transcripts identified as DEGs by the genome approach, all of them showing the same direction of expression (Additional file 2: Table S2). PCA for the 561 DEGs identified by both approaches showed that the biological differences between the dimorphic seeds are much greater than the differences deriving from the references used (Fig. 6c). All samples from the same seed morphs clearly clustered together, independently of the sequence reference (transcriptome or genome). The remaining 972 transcripts (63%) of the 1533 transcriptome DEGs did either not pass the 50% coverage cut-off (405/1533), only had a hit in one direction of the reciprocal BLAST (122/1533), their reciprocal hit was not a DEG in the genome (197/1533) or they did not produce any significant alignment at all (248/1533). Hence, approximately 40% of the DEGs from the de novo transcriptome assembly are equivalent to the DEGs found when a genome reference is available, and 60% of the DEGs were either fragmented or could not be clearly paired up with a gene model. This indicates that data for individual genes might not always be available when working with de novo transcriptome differential expression analysis. In cases like this, it might be important to perform other analyses that study the changes of global functions occurring in the samples, such as Gene Ontology bias. To verify the robustness of the expression pattern between the dimorphic seeds, we performed qRT-PCR on a selection of DEGs with varying levels of RPKM values in an independent biological experiment (Additional file 3: Figure S3). Despite the fact that the qRT-PCR results are derived from a completely independent experiment with different RNA samples, the expression patterns were confirmed for eight of the ten selected DEGs.

### Gene ontology analysis

**Fig. 7:**
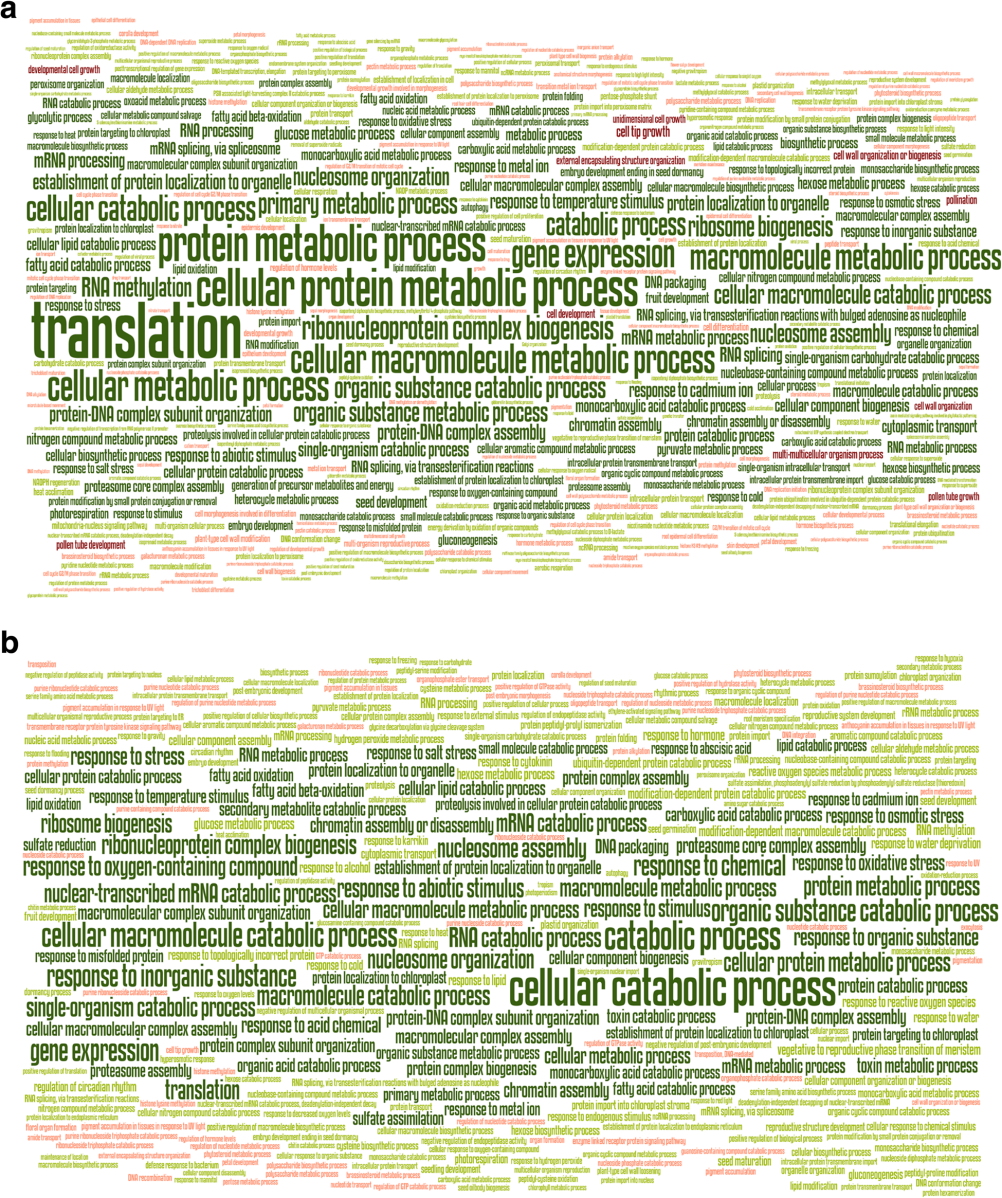
GO term word clouds of genome and transcriptome DEGs. Word clouds showing significantly over-represented (green) and under-represented (red) Biological Process terms for the genome DEGs (**a**) and the transcriptome DEGs (**b**). Word height is proportional to -log_10_(q-value), significantly over-represented GO-terms are coloured green (q < = 0.0001 dark green, q > 0.0001 light green) and under-represented GO-terms are coloured red (q < = 0.0001 dark red, q > 0.0001 light red)

The number of GO terms associated with the genome and the de novo transcriptome, for all transcripts, for the DEGs and for the overlap between both approaches is summarized in Table 1 (and in more detail in Additional file 2: Table S5–S6) and is referred to as a GO-presence list. When comparing (Comparison 4, Fig. 2) what is shared between the GO-presence list of the reference genome and the de novo transcriptome (All Transcripts Overlapping GO terms from Table 1; using Fisher’s exact test with an fdr corrected *p* value of 0.05), only 12 out of 5584 GO terms were shown to have significant differences in the number of transcripts associated to them (Additional file 2: Table S5). The GO-presence list of the DEGs (All DEGs Overlapping GO terms from Table 1) showed no significant differences at all between the genome and the transcriptome (Additional file 2: Table S6). Furthermore, having 1663 common GO terms present in the GO-presence lists of both DEG sets (Fig. 7) is a significant over-representation compared to the null hypothesis of selecting 1901 and 2191 GO terms randomly (Chi squared test, *p* = 2.2e-16). This suggests a biological signal, supporting that functional analysis of GO terms by transcriptome de novo assembly resembles the data derived by genomic analysis.

**Table 1:** Summary of GO terms associated with both the genome- and transcriptome-derived transcripts and respective DEG sets

		Transcriptome	Genome
All Transcripts	Total number	34,784	23,594
	Number with GO terms	18,845 (54%)	18,320 (78%)
	GO terms per transcript^a^	7.1	7.9
	Amount of GO terms	6091	6080
	Overlapping GO terms	5584	
All DEGs (M^+^ + NM)	DEGs	1533	1876
	Amount of GO terms	1901	2191
	Overlapping GO terms	1663	
NM-high^b^	DEGs	745	998
	Amount of GO terms	1427	1673
	Overlapping GO terms	1256	
NM-low^c^	DEGs	788	878
	Amount of GO terms	1085	1185
	Overlapping GO terms	880	

^a^Average including only transcripts with at least 1 GO term

^b^DEGs where transcript is more abundant in NM dry seed than M^+^ seed

^c^DEGs where transcript is less abundant in NM dry seed than M^+^ seed

For both the 1256 overlapping GO-terms of the DEGs GO-presence lists with higher abundance in NM (NM) seeds (“NM-high”) and 880 overlapping GO-terms of the DEGs GO-presence lists of with lower abundance in NM seeds (“NM-low”), none had significantly different quantities of underlying transcripts. The numbers and overlap of significantly over- and under-represented GO-terms of each class (Biological Process (BP), Molecular Function (MF) and Cellular Component (CC)) for all, NM-high and NM-low DEGs derived from the two approaches are summarized in Additional file 2: Table S7 and in more detail in (Additional file 4: Table S8) and are referred to as GO-bias lists. Overall, the NM-high and NM-low BP GO-bias lists are quite different. In the reference genome approach, NM-high has 340 unique BP terms, NM-low has 137 unique BP terms in the respective GO-bias list, with only 58 BP terms overlapping between both sets. Some of the most significant overlapping BP terms belong to high-level categories, such as ‘protein metabolic process’ and ‘gene expression’ (comprehensive lists of GO terms associated with the DEG sets are provided in Fig. 7). In agreement with this, ribonucleoprotein complex is the most significantly over-represented CC term in the genome approach, and structural constituent of ribosome is the most significantly over-represented MF term (Additional file 4: Table S8).

Many of the GO-terms found to be significantly over-represented and under-represented using the transcriptome approach were also found with the genomic approach: Out of the 321 BP terms found to be significantly over (255) and under (66) represented in the transcriptome-derived DEG set (GO-bias lists) (Fig. 7b and Additional file 4: Table S8), 258 (80%) were also found to be the same in the genome-derived DEG set (GO-bias lists) (Fig. 7a and Additional file 4: Table S8. On average, approximately 80% of the significantly over- and under-represented GO terms of the transcriptomic DEG sets (GO-bias lists) were also reported using the genomic approach. So, in comparison to the 40% overlap of DEGs on a gene-transcript pair level, we found a much higher overlap of differentially expressed functions between the *Ae. arabicum* M^+^ and NM dimorphic seeds using GO term bias analysis, even though some of the genes involved in these functions are missing in the transcriptome DEG dataset. The genomic approach reports on average 37% more GO-terms to be significantly over- or under-represented, which can be explained by the 22% more DEGs and 10% more GO-terms per gene. Though a transcriptome de novo assembly approach gives less information, the information that is given overlaps very well with a genome-based approach. Taken together, this finding supports the view that analysis of GO terms by transcriptome de novo assembly is a useful tool when no genome is available, and resembles the data derived by genomic analysis.

### DEG analysis of mature dimorphic *Ae. arabicum* seeds

The most significantly over-represented BP terms unique to the NM-high DEGs GO-bias list (transcripts with a higher abundance in NM seed compared to M^+^ seed) include mRNA metabolic process, mRNA processing and response to stimulus. On the other hand, the most significantly over-represented BP terms unique to the NM-low DEGs GO-bias lists (transcripts with a lower abundance in NM seed compared to M^+^ seed) are translation, ribosome biogenesis and nucleosome assembly (Additional file 4: Table S8). This is also reflected in the CC and MF terms, with the nucleus CC term and RNA binding MF term being among the most significantly over-represented terms in the NM-high DEGs (GO-bias list) and the structural constituent of ribosome MF term and ribosome CC term being among the most significantly over-represented terms in the NM-low DEGs (GO-bias lists; Additional file 4: Table S8). Thus, it is generally indicative that the transcriptome of the M^+^ “dry” mature seed morph transcriptome may be relatively more oriented towards translation of RNA and chromatin assembly, whereas the NM “dry” mature seed morph transcriptome may be more oriented to post-transcriptional processing of RNA. It is possible that these differences may reflect the stage which was sampled – the dry seed. Thus, transcriptomic differences may be due to differences in the stage of seed development or maturation the seed morphs have reached before desiccation. For this reason, we put the transcriptomic differences between *Ae. arabicum* NM and M^+^ seed in context of the well-studied seed development and maturation of *A. thaliana*.

**Fig. 8:**
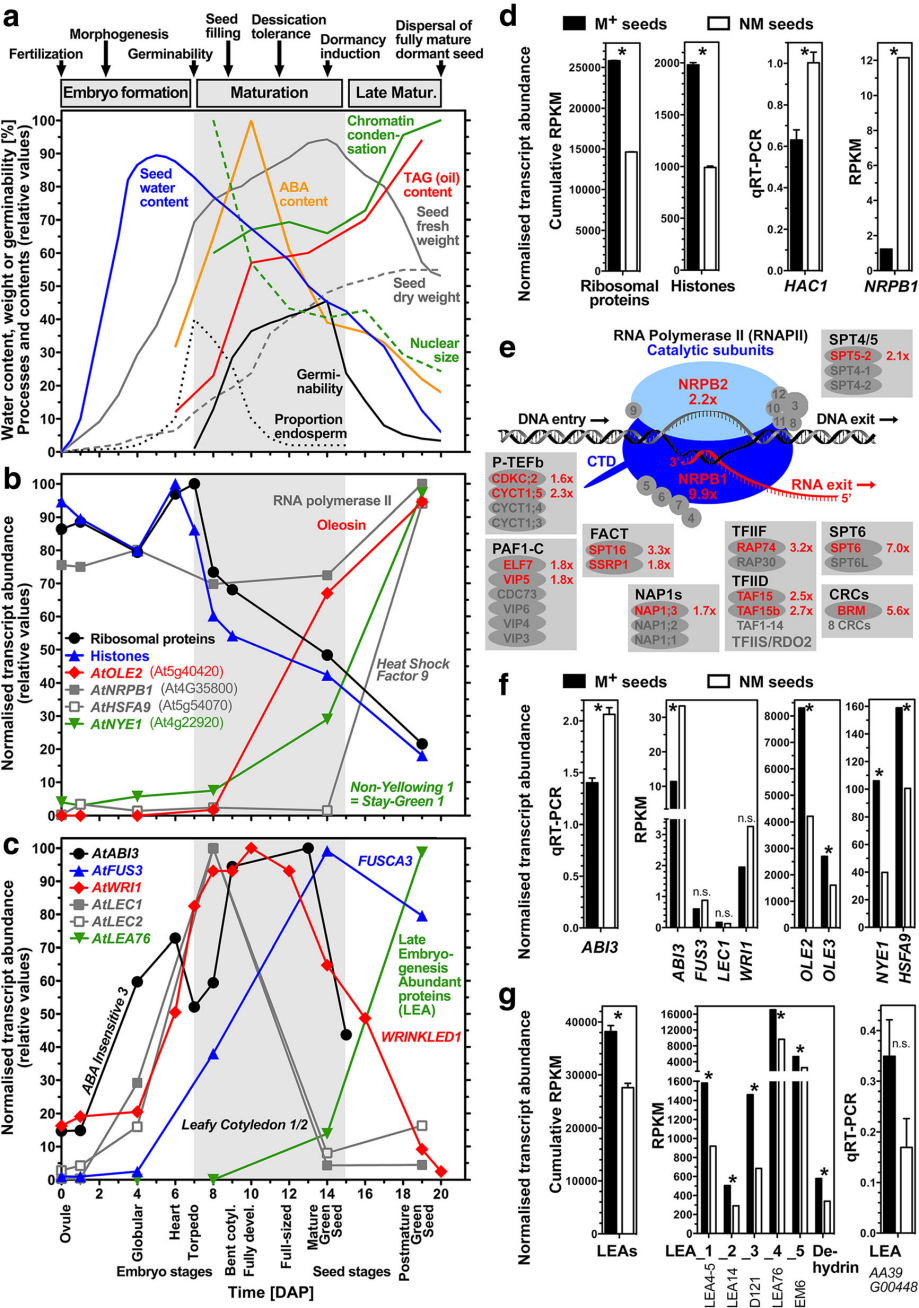
Key processes and differentially expressed genes (DEGs) differ between *Ae. arabicum* M^*+*^ and NM seeds. **a** Timing of key processes during development and maturation of *A. thaliana* seeds. Dormancy and desiccation tolerance coincides with changes in water, abscisic acid (ABA) and triacylglycerol (TAG) contents, seed weight, nuclear size and chromatin condensation, endosperm proportion and germinability; Data from [32, 41, 55]. **b** Selected *Ae. arabicum* DEG putative ortholog expression during *A. thaliana* seed development and maturation. Cumulative transcript abundances for *A. thaliana* putative orthologs of *Ae. arabicum* 21 histone and 119 ribosomal protein genes (Additional file 3: Figure S4); individual abundances for RNA polymerase II large subunit (*AtNRPB1*), oleosin *AtOLE2* (seed storage), heat shock factor *AtHSFA9* (longevity), and *AtNYE1* (chlorophyll degradation); data from Arabidopsis eFP browser [74] and [29–31]. **c** Expression of late embryogenesis abundant (*LEA*) proteins, seed maturation master regulators (*AtLEC1*, *AtLEC2*, *AtABI3, AtFUS3*) and *WRINKLED1* (*AtWRI1*), a transcription factor associated with enhanced fatty acid and TAG biosynthesis during *A. thaliana* seed maturation; data from Arabidopsis eFP browser and [29–31, 58]. **d** Expression of selected *Ae. arabicum* DEGs for ribosomal proteins, histones, *NRPB1* (RNAseq) and histone acetyltransferase *HAC1* (qRT-PCR) in M^*+*^ and NM seeds. Cumulative RPKM values presented for 21 histone and 119 ribosomal protein genes of *Ae. arabicum* (Additional file 3: Figure S4). A * indicates a significant difference between M^+^ and NM seeds based on using a t-test (*p* < 0.05); n.s. means ‘not significant’. **e** Expression of RNA polymerase II complex and associated factors [50, 51] that mediate transcription including initiation, elongation and processing of transcripts in *Ae. arabicum* dry seed morphs. Red text indicates factor identified as NM-high DEG with expression ratio (NM / M^*+*^) indicated. Note core NRPB1/2 transcript abundance and most factors are several-fold higher in NM seeds. **f** Seed maturation master regulators expression (RNAseq, *ABI3* also by qRT-PCR), oleosins, *NYE1* and *HSFA9* in dry M^*+*^ and NM *Ae. arabicum* seeds. **g** Selected *Ae. arabicum LEA* expression in dry M^*+*^ and NM seeds (RNAseq and qRT-PCR). The presented dehydrin is the putative ortholog of At4G39130. Error bars indicate mean ± SEM for qRT-PCR experiments. For the plotted RPKM values of single genes from the RNAseq data we used the result of the DEG detection pipeline (edgeR + NOISeq + DESeq2) as the indicator of significance

The *Ae. arabicum* M^*+*^ seed morph as well as *A. thaliana* seeds are both dispersed from dehiscent fruits and seem to resemble each other in terms of morphology and physiology [3]. In Fig. 8, we compare the expression of selected *Ae. arabicum* key DEGs (which differ between the dimorphic M^*+*^ and NM seeds, selected based on the prominent GO terms and genes with importance to seed development and maturation) with the expression of their putative orthologs derived from published transcriptomes of developing and mature *A. thaliana* seeds [29–31]. During the *A. thaliana* seed maturation and late maturation phases desiccation tolerance and dormancy are established in parallel with drying resulting in the low-hydrated dispersed seed state (Fig. 8a) [32, 33].

For the dry mature *Ae. arabicum* dimorphic seeds, we found that the abundance of at least 119 (reference approach) and 113 (de novo approach) ribosomal protein transcripts were 1.5- to 3-fold higher in M^*+*^ seeds as compared to NM seeds (Fig. 8d, Additional file 3: Figure S4a). This seems to be a general pattern as there were no ribosomal protein genes with higher transcript abundances in NM seeds. The abundance of the putatively orthologous transcripts of these DEGs decreased during *A. thaliana* seed maturation (Fig. 8b). A genome-wide analysis of ribosomal protein gene expression during *A. thaliana* and *Brassica napus* seed maturation revealed the same temporal pattern [30, 34]. During maturation, ribosomal activity is required for processes such as seed storage compound accumulation which deceases upon late maturation drying. In dry seeds, ribosomes are mainly present in the monosome form [35]. Ribosomal profiles change with polysomes being formed during seed germination and subsequent seedling growth. Interestingly, during these processes, differential expression of ribosomal protein genes occurs and may affect ribosome composition and thereby the selection of translated mRNAs [31, 35–37]. 35–40% (reference approach) and ~ 30% (de novo approach) of the ribosomal protein genes in M^*+*^ seeds show approximately 2-fold higher transcript abundances, which suggests that they dry out earlier during late maturation as compared to NM seeds. Considering their overall decrease over time during seed maturation (Fig. 8b), this would explain the higher abundance of transcripts for ribosomal protein genes in dry M^+^ seeds. Alternatively, M^+^ seeds could have a higher translational activity with a higher ribosome per seed content. In the latter case, we would also expect elevated rRNA biosynthesis in the larger M^+^ seeds as compared to the smaller NM seeds. This is however not the case, as evident from the rRNA amounts estimated by filtering during the RNA-seq workflow (Figs. 2 and 3). We therefore conclude that the higher transcript abundance of a large number of ribosomal protein genes in M^*+*^ seeds seems to be due to faster drying of M^+^ seeds during late maturation. This conclusion is also consistent with the DEG patterns for histones and other genes as discussed later. We propose that the earlier drying out may preserve the mature M^+^ seeds in a state with higher ribosome content and translational activity compared to the mature NM seeds. The distinct states are consistent with the distinct germination and dormancy behavior of the dimorphic *Ae. arabicum* seeds [3].

The NM-low DEGs of the reference approach related to nucleosome assembly include 21 *Ae. arabicum* histone genes, including seven H4, five H3, four H2B, five H2A, but no H1 homolog of *A. thaliana* histone variants. For the dry mature *Ae. arabicum* dimorphic seeds, we found that the abundance of these histone transcripts were 1.5- to 4-fold higher in M^*+*^ seeds as compared to NM seeds (Fig. 8d, Additional file 3: Figure S4b). The NM-low DEGs of the de novo approach related to nucleosome assembly include nine histone genes, including four H3, two H2B, three H2A, with transcript abundance of 1.5- to 4-fold higher in M^+^ seeds as compared to NM seeds. Like the ribosomal protein DEGs, the transcript abundance of the *A. thaliana* histone homologs decreased during seed maturation (Fig. 8b). As with the ribosomal protein DEGs, the approximately 2-fold higher histone transcript abundance in M^+^ seed could be due to faster drying of M^+^ seeds during late maturation. However, as these DEGs represent only ca. 20% of the histones they may serve specific roles which define distinct processes in the dimorphic *Ae. arabicum* seeds. Differential expression of histone variants is linked to DNA replication and transcriptional regulation in response to developmental or environmental cues [38–40]. Histones are major components of chromatin, the protein-DNA complex involved in DNA packaging, chromatin remodeling and heterochromatin formation. *A. thaliana* seed maturation is characterized by nuclear size reduction and increased chromatin condensation (Fig. 8a) [41]. Chromatin condensation and heterochromatin formation involves the expression of specific histone H2B, H2A, and H3 variants [42–44], some of which we found to be *Ae. arabicum* DEGs with higher transcript abundance in M^*+*^ compared to NM seeds (Fig. 8d). In contrast to those histone transcripts which are NM-low DEGs, genes which modify histones and facilitate transcription and RNA processing were found among the NM-high DEGs. Several genes encoding histone acyetyltransferases, deacetylases, and methyltransferases are among the NM-high DEGs, including for example putative orthologs of *A. thaliana HAC1* (At1g79000), *HAC12* (At1g16710), *HDA19* (At4g38130), *EFS* (At1g77300) and a SET7/9 family protein (At4g17080) (Fig. 8d, Additional file 3: Figure S4b), with HAC1, HAC12 and EFS putative orthologs being classified as transcriptional regulators by TAPscan (Additional file 2: Table S3). The NM-high DEGs of the de novo approach included *HAC1* (At1g79000), *HAC12* (At1g16710) and *EFS* (At1g77300), with HAC12 and EFS putative orthologs being classified as transcriptional regulators by TAPscan (Additional file 2: Table S4). These histone modifications are involved in regulating seed maturation and dormancy in response to environmental cues [43]. EFS for example is known to inhibit seed germination [45], HDA19 to repress seed maturation genes [46], and HAC1 to affect seed production and germination [47].

The absence of histone H2B mono-ubiquitination in the *A. thaliana hub1* and *hub2* mutants leads to altered chromatin remodeling and reduced seed dormancy [43, 44, 48], but the *HUB1/2* putative orthologs were not among the *Ae. arabicum* NM-high and NM-low DEGs. *HUB1/2* interacts with the FAcilitates Chromatin Transcription (FACT) complex, consisting of the SSRP1 and SPT16 proteins, for which mutants exhibit reduced seed production [49, 50]. The FACT complex is a histone chaperone that assists the progression of transcribing RNA polymerase II (RNAPII) on chromatin templates by destabilizing nucleosomes. The transcript abundance of the RNAPII catalytic subunit *NRPB1* increases during the late seed maturation of *A. thaliana* (Fig. 8b). Interestingly, putative *Ae. arabicum* putative orthologs of both RNAPII catalytic subunits were among the NM-high DEGs of the reference approach, with *NRPB1* approximately 10-fold and *NRPB2* 2-fold higher in NM seeds (Fig. 8d, e). *NRPB1* and *NRPB2* were also present with similar expression values in the NM-high DEGs of the de novo approach. Further to this, several key components of the RNAPII elongation complex [50–52] were also among the NM-high DEGs of both approaches, including transcripts of subunits of almost all known factors known to be involved in regulating RNAPII-mediated transcription initiation, elongation and processing (Fig. 8e, Additional file 3: Figure S4b). In contrast to this, there were no such factors among the NM-low DEGs. Mutants for several of these key components are known for their developmental phenotypes including seed germination and dormancy traits [43, 48, 49, 52, 53]. Moreover, several other transcripts in downstream RNA processing were also among the NM-high DEGs of both approaches. Examples for this include factors with RNA binding, splicing and helicase activity (Additional file 3: Figure S4b). Among them is SMG7 (detected in both approaches) which is involved in nonsense-mediated mRNA decay (NMD) and regulates seed number in *B. napus* [54]. Taken together, these findings support the view that the transcriptome of NM seeds seems to be geared towards transcription which is important for dormancy and persistence. In contrast to this, seed maturation of M^+^ seeds lead to a dry seed transcriptome in which translation is most dominant and is also most important during germination.

### Dimorphic *Ae. arabicum* seeds differ in their maturation programmes

Seed-related processes were also amongst the BP terms significantly over-represented in the DEGs (GO-bias list), with the terms embryo development, fruit development, seed development and seed dormancy common to both the NM-high and NM-low DEG list (GO-presence list) (GO terms for each list can be found in Additional file 4: Table S8). However, the BP terms seed maturation, seed germination and seedling development were specific to the NM-high DEG GO-presence list. Additionally, the more specific BP terms positive regulation of seed maturation and negative regulation of seed germination were also identified in the NM-high DEG list. On the other hand, the term seed oil body biogenesis was only identified in the NM-down DEG GO-presence list. Thus, it appears that the M^+^ and NM seed morphs differ in their expression of genes which determine seed traits during maturation. Seed maturation is associated with abscisic acid (ABA) regulated storage reserve accumulation such as oil (triacylglycerol, TAG) which requires gene expression [33, 55–58]. To achieve this fatty acid and TAG biosynthesis genes encoding proteins such as long chain acyl-CoA synthetase (LACS) and acyl-CoA:diacylglycerol acyltransferase (DGAT) are upregulated during *A. thaliana* seed maturation [59]. The TAGs are then transferred and accumulated into oil bodies which are covered on their surface with oleosins. Oleosins are the most abundant proteins found in the seed proteomes of oilseeds [57, 58]. Oleosin gene expression is also upregulated during *A. thaliana* seed maturation (Fig. 8b), but transcript abundances subsequently decline at the end of late maturation [57]. Their roles include to control oil body dynamics, size, and total oil accumulation during seed maturation. Interestingly, while putative orthologs of *A. thaliana LACS7*, *DGAT1*, a fatty acid alcohol dehydrogenase and a lipid transporter are among the NM-high DEGs of the reference approach (Additional file 3: Figure S4b), two oleosin homologs, *OLE2* and *OLE3*, are among the NM-low DEGs (Fig. 8f). In the de novo approach, putative orthologs of *LACS7* and *OLE2* are present among the NM-high and NM-low DEGs respectively, while the *DGAT1* putative ortholog was not detected as DEG and no *OLE3* homolog could be identified. That oleosin and TAG biosynthesis genes are in distinct DEG groups may either be due to distinct regulation during late seed maturation with TAG biosynthesis still up while oleosin expression is declining, or due to more profound differences between the dimorphic seeds in their maturation processes.

Four master regulators of seed maturation have been identified in *A. thaliana*: *ABSCISIC ACID INSENSITIVE3* (*ABI3*, At3g24650), *FUSCA3* (*FUS3,* At3g26790), *LEAFY COTYLEDON2* (*LEC2*, At1g28300), and *LEAFY COTYLEDON1* (*LEC1,* At1g21970) [33, 59, 60]. Whilst *LEC1* encodes the HAB3 subunit of a CCVAAT-box binding TF, ABI3, FUS3, and LEC2 are TFs with a B3 DNA binding domain. Corresponding TF classification was detected in the *Ae. arabicum* putative orthologs using TAPscan (Additional file 2: Table S3). In the de novo approach, orthologs of the ABI3/VP1 TFs *ABI3* and *FUS3* could be identified, with only *FUS3* being identified by TAPscan, probably because of the shorter length of the transcriptome based protein (577aa) vs. the reference based one (701aa) (Additional file 2: Table S4). These four master regulators control seed maturation including fatty acid and TAG biosynthesis, as well as oleosin expression and oil body formation. Enhancement of fatty acid and TAG biosynthesis by these master regulators is achieved, at least in part, by interaction of the *WRINKLED1* (*WRI1*, At3g54320) TF of the AP2/EREBP family [56, 58–61]. The temporal transcript patterns of these genes during *A. thaliana* seed maturation is depicted in Fig. 8c. Consistent with the *Ae. arabicum* fatty acid and TAG biosynthesis genes being among the NM-high DEGs, the putative *Ae. arabicum ABI3* ortholog is among the NM-high DEGs in the reference approach, with a putative *WRI1* ortholog also tending towards higher expression in NM seed (Fig. 8f). It should be noted that the *WRI1* transcript (TR24803|c0_g1_i1) is not represented by a gene model in the current genome version, demonstrating that occasionally the de novo transcriptome approach might outcompete the genomic approach. However, *FUS3* and *LEC1* are expressed roughly equal in dry M^+^ and NM seeds (Fig. 8f). Also, if earlier drying of M^+^ seeds is the only difference compared to NM seeds, *WRI1* and *ABI3* should be among the NM-low DEGs because their transcript abundances decline in *A. thaliana* during late maturation (Fig. 8c). It therefore seems that M^+^ seeds not only dry out earlier, but also mature faster as compared to NM seeds. That M^+^ seed maturation is faster is further supported by the finding that the *Ae. arabicum* NM-low DEG list of the reference approach contains the putative orthologs of *NON-YELLOWING1/STAY-GREEN1* (*NYE1/SGR1*, At4g22920), *HEAT SHOCK TRANSCRIPTION FACTOR9* (*HSFA9*, At5g54070) and of several Late Embryogenesis Abundant (LEA) protein genes which are upregulated during *A. thaliana* seed maturation (Fig. 8b, c) and are among the NM-low DEGs (Fig. 8f, g). The same findings were made using the de novo approach except that the *HSFA9* was not in the NM-low DEG list, but only trended towards lower expression in NM seeds. Efficient chlorophyll degradation during late seed maturation, in part mediated by the NYE1 protein, is critical for seed quality, longevity (storability), dormancy and germination properties [62]. During seed maturation, ABI3, through HSFA9, induces the accumulation of a subset of heat shock proteins (HSP) that contribute to seed longevity by protecting protein molecules and structures in the dry state [33, 63]. Among the *Ae. arabicum* DEGs, there are indeed *HSF9* and two other HSFs and several HSPs, but different HSPs are expressed in either a NM-low or a NM-high specific manner (Fig. 8f, Additional file 3: Figure S4b). A more distinctive pattern was obtained for the LEA proteins which were primarily found among the *Ae. arabicum* NM-low DEGs (Fig. 8g), supporting the view that M^+^ seeds may mature faster and that M^+^ and NM seeds may differ in their longevity.

Accumulation of LEA proteins is a landmark of seed maturation and several accumulate only during late maturation drying [33]. The 51 LEA protein encoding genes identified in *A. thaliana* cluster into 9 groups including LEA_1 to LEA_5, Seed Maturation Proteins (SMP) and dehydrins [64]. In the reference approach we found 13 putative LEA orthologs from all these groups in the *Ae. arabicum* NM-low and only two in the NM-high DEGs list (Fig. 8f, Additional file 3: Figure S4b). In the de novo approach, six LEA homologs were amongst the NM-low and only one in the NM-high DEGs list. The cumulative LEA transcript abundances were higher in M^+^ compared to NM seeds, and the known most abundant LEA genes followed this pattern (Fig. 8f). Among them are the putative orthologs of *A. thaliana* LEA_1 *LEA76* (At5g06760), LEA_4 (At3g15670), LEA_5 *EM6* (At2g40170), the SMP *RAB28*, and dehydrins which are also most abundant in mature *A. thaliana* seeds [65]. The *A. thaliana* mutant *em6–1* is altered in seed hydration and desiccation tolerance during seed maturation [66]. LEA proteins are highly hydrophilic and intrinsically unstructured, and act by protecting proteins and enzyme activities in the desiccated state which, together with HSPs, may lead to maintaining seed longevity during dry storage [33, 63, 64]. In addition to their higher LEA transcript abundance (Fig. 8g), in both approaches, M^+^ seeds also have higher transcript abundances of enzymes involved in detoxifying Reactive Oxygen Species (ROS) such as superoxide dismutase (SOD) and glutathione-*S*-transferase (GST) (Additional file 3: Figure S4b). ROS are produced during a number of seed related processes: with potentially deleterious effects during seed maturation, desiccation, ageing and germination; but also acting by controlling dormancy and germination [63, 67, 68]. Thus, the two seed morphs may differ in mechanisms by which seed longevity and dormancy are established and regulated. Whilst the GO term ‘hormone metabolic process’ was amongst 137 BP GO terms significantly under-represented in the reference approach DEGs (GO-bias list), the putative orthologs of genes involved in ABA and gibberellin signaling (*XERICO*), ethylene biosynthesis (*S-adenosylmethionine synthetase*, *SAMS3*) and signaling (*EIN3-binding F-box protein*, *EBF1*), and auxin and brassinosteroid signaling (*Auxin Response Factor 2*, *ARF2*) are amongst the DEGs (Additional file 3: Figure S4b), with all but *XERICO* also being among the de novo approach DEGs. The presence of these genes is consistent with previously observed differences in seed development and dormancy (described further in Additional file 3: Figure S5).

## Conclusions

RNA-seq analysis of *Ae. arabicum* M^+^ and NM dry seed transcriptomes using either a de novo assembled transcriptome approach or reference genome guided approach showed only a modest overlap in the DEGs identified, but much greater consistency in the GO terms identified. Thus, using global functional annotations such as GO terms, the de novo assembled transcriptome approach would result in similar conclusions being drawn from the data compared to the reference genome approach. Studying seeds, which are a well characterized biological system, allowed us to identify many well studied genes and put them into context using both a de novo assembled transcriptome approach and a reference genome guided approach. This highlights the potential usefulness of de novo transcriptome assembly in the study of species that do not have a reference genome. With the decreasing costs of RNA-seq one should aim for using at least three replicates, potentially bridging the gap between a de novo assembly and reference genome guided approach even further. However, our results also highlight the limitations of de novo transcriptome analysis. Namely, if the goal is to pinpoint the DEGs underlying a trait, then reference based assemblies perform better.

Major differences in the seed morph transcriptomes were highlighted by GO analysis. In particular, genes associated with translation and histone assembly were more abundant in the less dormant M^+^ dry seed, whereas genes associated with transcription and mRNA processing were more abundant in the more dormant NM dry seed. By putting the M^+^ and NM dry seed transcriptomes in the context of transcriptomes from developing and maturing *A. thaliana* seeds, it was indicated that M^+^ seeds may both desiccate earlier (M^+^ has higher histone and ribosomal protein expression) and mature faster than NM seeds (compared to NM, M^+^ seed have higher expression of genes that increase with maturation, such as homologs of LEAs, *NYE1* and *HSFA9*, and lower expression of genes that decrease during maturation such as *ABI3* and *WRI1*). The differences identified align with the known development and germination behaviour of the two seed morphs, but hint at other differences such as in longevity mechanisms (LEAs, ROS detoxification). However, the difference in longevity of M^+^ and NM seed are so far unknown. It would also be valuable to study how the differences in dry seed lead to differences in transcription and germination physiology in the imbibed dimorphic seeds.

## Methods

### Plant material and RNA extraction

*Aethionema arabicum* (L.) A.DC. accession 0000309 (collected from Turkey and obtained from Kew’s Millennium Seed Bank, UK) and ES1020 (collected from Turkey and obtained from Eric Schranz, Wageningen) [3] plants were grown on soil under long-day conditions (16 h light/20°C and 8 h dark/18°C). Freshly matured seeds from dehiscent (harboring M^+^ seeds) and indehiscent (harboring NM seeds) fruits derived from several plants were harvested. Two replicates of 20 mg fresh dry M^+^ and NM seeds, resulting in four samples in total, were pulverized in liquid N_2_ using a mortar and pestle. RNA extraction was performed according to [69]. RNA integrity was checked by gel electrophoresis (Additional file 3: Figure S6) followed by quantity and purity determination with a Nanodrop spectrophotometer ND-1000 (Peqlab) showing sufficiently low levels of degradation for RNAseq and OD ratios of at least 2 (260/280 nm) and 1.8 (260/230 nm).

### RNA-seq library preparation and sequencing

RNA libraries were prepared following instructions of the TruSeq™ RNA library prep kit (Illumina) using oligo-dT-based mRNA selection. Libraries were sequenced using a HiSeq-2000 sequencer (Illumina) generating 100 bp single-end reads.

### RNA-seq data trimming and filtering

The raw RNA sequences were processed with trimmomatic [15] (ILLUMINACLIP:adaptors:2:20:8, SLIDINGWINDOW:4:15, TRAILING:15, HEADCROP:12, MINLENGTH:20) to remove poor quality stretches and adaptors. Poly-A and Poly-T tails were removed using PrinSeq [16]. To reduce the complexity of the dataset prior to mapping our reads to the genome/transcriptome rRNA, mitochondrial and chloroplast sequences were filtered. Since *Ae. arabicum* sequences for rRNA, mitochondria and chloroplast were not available in public repositories, sequences from closely related and well annotated *A. thaliana* were used. GSNAP version 2016–11-07 [17] with default settings was used to map the reads against the chloroplast (GenBank: AP000423.1), mitochondria (GenBank: Y08501.2) and rRNA (GenBank:X52320.1) sequences from *A. thaliana*.

### De novo transcriptome assembly

Prior to the de novo transcriptome assembly, redundant duplicate reads, i.e. reads with the exact same length and sequence, were removed since they might constitute PCR artefacts. The trimmed, filtered and de-duplicated reads were assembled into a transcriptome using Trinity [14] with default settings. For each isoform group, the longest transcript was chosen as representative and its longest open reading frame was translated into protein using a custom python script.

### Evaluation of assembly and comparison to genome

Genome scaffolds and accompanying GFF file of *Ae. arabicum* genome version 2.5 [5] was obtained from CoGe (genome id23428, https://genomevolution.org/coge/OrganismView.pl?gid=23428). The CDS of each gene was translated into proteins using the R package biostrings version 2.32.0. The completeness of the assembled transcriptome and the available genome of *Ae. arabicum* was evaluated using the Benchmarking Universal Single-Copy Orthologs tool BUSCO v3.0.1 [23] and their accompanying dataset of 1440 plant orthologs (embryophyta *odb9*). To investigate how well the assembled transcripts represented and paired up with the existing gene models from *Ae. arabicum* genome version 2.5, reciprocal BLAST (version 2.2.29+, [70]) searches were carried out. Reciprocal best hits (RBH) with a minimum query and subject coverage of 50% each were considered as a match and selected for comparison.

### Read mapping and feature counting

Processed reads were mapped against the assembled transcriptome and the *Ae. arabicum* genome version 2.5 using GSNAP with default settings. Reads that mapped to multiple positions in the genome were discarded and only uniquely mapped reads were kept. Mapped reads per feature were counted using HTSeq-count (version 0.6.1 [25]) with the options “–s no –t gene –m union”. For the transcriptome a custom GFF was generated with one feature for each transcript, while for the gene models the GFF mentioned above was used. The average coverage was calculated using the genome reference. The total amount of mapped reads (all libraries) for each gene was multiplied by the read length (83) and divided by gene length (Additional file 2: Table S1).

### Differential gene expression analysis pipeline

Differentially expressed genes were identified using R [71] and the Bioconductor packages DESeq2 version 1.14.1 [19], edgeR version 3.16.5 [18] and NOISeq version 3.16.5 [20]. It is recommended to discard features with low counts for edgeR DEG analysis, so only genes with at least 10 read counts when summing up all the sample counts were selected for edgeR. Default parameters were used for DESeq2, edgeR (classic approach, “exactTest”) and NOISeq with normalization method relative log expression for DESeq2, trimmed mean of M values for edgeR and RPKM for NOISeq. DESeq2 and edgeR make use of Benjamini-Hochberg [72] adjusted *p*-value (q-value) cut offs which were set to 0.001. For NOISeq, which uses probabilities of differential expression, a cutoff value of > 0.9 was used. This is higher than the recommended 0.8 but has been shown to overlap well with experimental array data, representing a conservative (specific) selection of DEGs [28]. The overlap (strict consensus) of the three packages’ outputs was used for further analysis.

### Principal component analysis of expression values

To compare the feature counts of the two approaches (de novo transcriptome and reference genome), PCAs were carried out using the built in R package prcomp. RPKM normalized expression values of the 6745 paired de novo transcripts and reference genes were calculated and used as input, as well as the 561 DEGs identified by both approaches.

### Annotation and GO-bias

The transcripts of the genome and assembled transcriptome were blasted against the nr database of NCBI (nucleotide release 13-05-2015), UniProtKB/Swiss-Prot (protein release 10–2015) and TAIR 10 (proteins release 20,110,103). GO-terms were retrieved using BLAST2GO version 2.5 [21] in combination with the NCBI nr blast results. GO-bias, i.e. over/under-representation of GO-terms in defined sets of genes as compared to all genes, was calculated as in [73] using Fisher’s exact test with FDR correction [72]. Wordle (www.wordle.net) was used to build word clouds, with word height proportional to –log10(q-value), significantly over-represented GO-terms colored green (q < = 0.0001 dark green, q > 0.0001 light green) and under-represented GO-terms colored red (q < = 0.0001 dark red, q > 0.0001 light red). Transcripts of the genome and assembled transcriptome were screened for TAPs using the TAPscan pipeline [26].

### qRT-PCR analysis

For technical as well as biological validation of RNA-seq derived gene expression data, RNA was extracted from separate batches of dry fresh mature M^+^ and NM seeds (five biological replicates each) as described above, and quantitative RT-PCR analysis of selected candidate genes was performed as previously described [69]. As normalization factor the geometric mean of three reference genes, *Ae. arabicum* putative orthologs of *ACTIN2* (*ACT2*, AA26G00546), *POLYUBIQUITIN10* (*UBQ10*, AA6G00219) and *ANAPHASE-PROMOTING COMPLEX2* (*APC2,* AA61G00327) was used, which was found to show comparable stable expression in M^+^ and NM seeds (Additional file 3: Figure S7). Primers for qRT-PCR are listed in (Additional file 2: Table S9).

## Additional files

Additional file 1: de novo transcriptome assembly. The 34,784 longest gene sequences from each Trinity gene cluster. (FA 28331 kb)

Additional file 2: Gene coverage calculation (**Table S1**), reciprocal best BLAST paring (**Table S2**), full annotation and RPKM tables for the genome method (**Table S3**) and transcriptome method (**Table S4**). Comparison of abundance of transcripts (genome method vs. transcriptome method) belonging to: the 5584 GO terms shared between both methods (**Table S5**); or the 1663 overlapping GO terms of the DEG sets (**Table S6**). **Table S7** shows a summary of significantly under- and over-represented GO terms associated with DEG lists. **Table S9** contains a list of primers used for qRT-PCR. (XLSX 10033 kb)

Additional file 3: **Figure S1.** PCA of RPKM values for 6745 paired transcripts (identified in both genome and transcriptome methods) by method and morphotype. **Figure S2.** RPKM levels (reference genome approach) of the overlapping DEGs as well as of the non-overlapping DEGs called by NOISeq, edgeR and DESeq2. **Figure S3.** Expression of selected DEGs measured by qRT-PCR. **Figure S4.** showing abundances of *Ae. arabicum* ribonucleoprotein transcripts (a) and transcripts from selected gene categories (b) and **Figure S5.** showing the pattern of expression of select hormonal signaling related genes during *A. thaliana* seed maturation. Assessment of RNA integrity and purity (**Figure S6.**) and validation of reference genes used for qRT-PCR normalization (**Figure S7.**) (DOCX 2738 kb)

Additional file 4: **Table S8.** Excel document containing GO term analysis output for BP, CC and MF classes and all DEG lists. (XLSX 347 kb)

## Abbreviations

ABA: Abscisic acid
ABI3: ABSCISIC ACID INSENSITIVE3
BP: Biological Process
BUSCO: Benchmarking Universal Single-Copy Orthologs
CC: Cellular Component
CDS: Coding sequence
DEGs: Differentially expressed genes
DEH: Dehiscent
DGAT: Acyl-CoA:diacylglycerol acyltransferase
FACT: FAcilitates Chromatin Transcription
FDR: False Discovery Rate
GFF: General feature format
GO: Gene ontology
HSFA9: HEAT SHOCK TRANSCRIPTION FACTOR9
HSP: Heat shock proteins
IND: Indehiscent
LACS: Long chain acyl-CoA synthetase
LEA: Late Embryogenesis Abundant
LEC1: LEAFY COTYLEDON1
LEC2: LEAFY COTYLEDON2
M^+^: Mucilaginous
MF: Molecular function
NM: Non-mucilaginous
NYE1/SGR1: NON-YELLOWING1/STAY-GREEN1
PCA: Principal component analysis
RNAPII: RNA Polymerase II
RNA-seq: RNA-sequencing
ROS: Reactive Oxygen Species
RPKM: Reads Per Kilobase per Million mapped reads
rRNA: ribosomal RNA
SMP: Seed Maturation Proteins
TAG: Triacylglycerol
TAPs: Transcription Associated Proteins
WRI1: WRINKLED1
