# Supplementary material for: Usability of reference-free transcriptome assemblies for detection of differential expression: a case study on Aethionema arabicum dimorphic seeds
Source: BMC Genomics. 2019 Jan 30;20:95. doi: 10.1186/s12864-019-5452-4 (PMC6354389; doi:10.1186/s12864-019-5452-4)
Supplement: Supplementary file 3 — Figure S1. PCA of RPKM values for 6745 paired transcripts (identified in both genome and transcriptome methods) by method and morphotype. Figure S2. RPKM levels (reference genome approach) of the overlapping DEGs as well as of the non-overlapping DEGs called by NOISeq, edgeR and DESeq2. Figure S3. Expression of selected DEGs measured by qRT-PCR. Figure S4. showing abundances of Ae. arabicum ribonucleoprotein transcripts (a) and transcripts from selected gene categories (b) and Figure S5. showing the pattern of expression of select hormonal signaling related genes during A. thaliana seed maturation. Assessment of RNA integrity and purity (Figure S6.) and validation of reference genes used for qRT-PCR normalization (Figure S7.) (DOCX 2738 kb) [file 12864_2019_5452_MOESM3_ESM.docx]

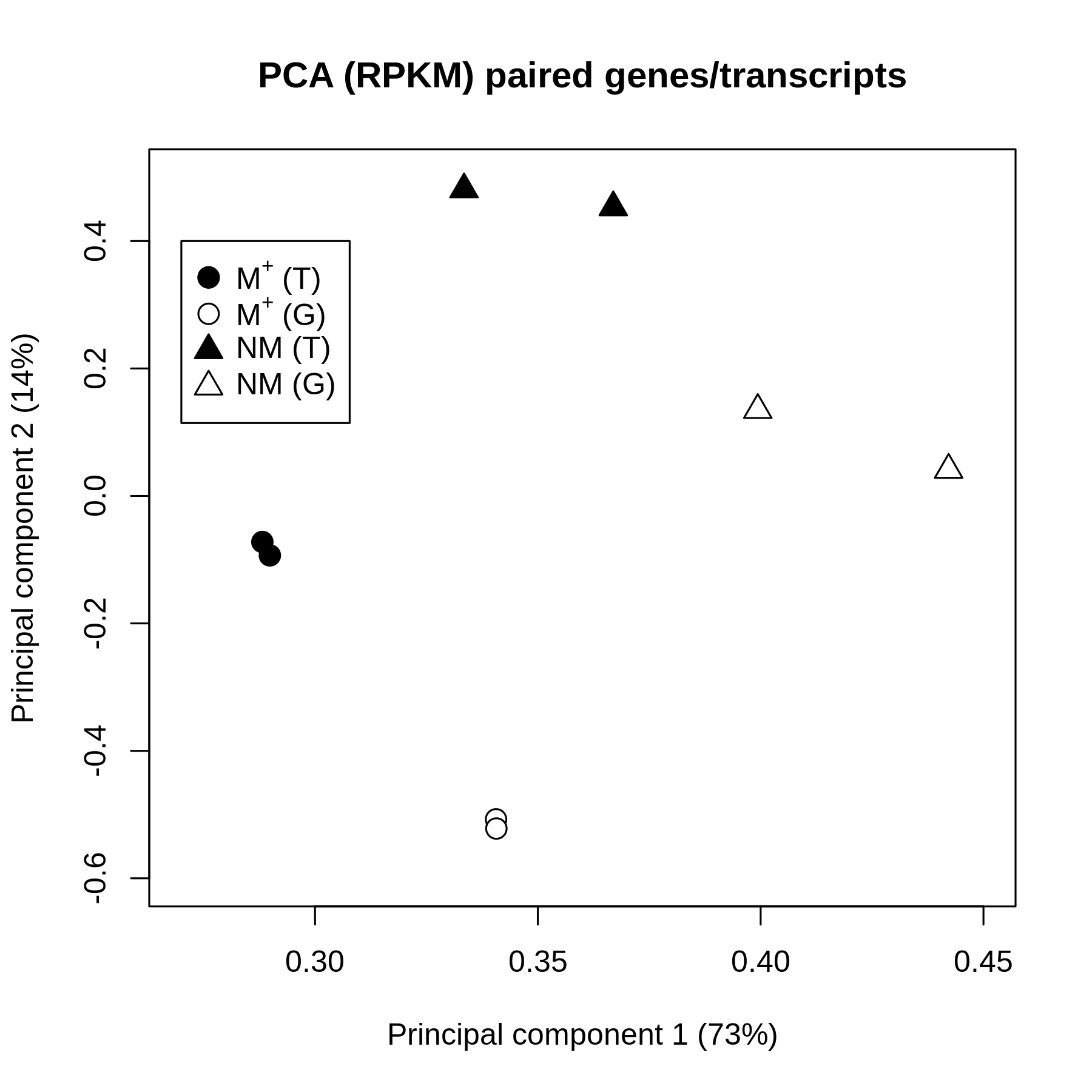


**Figure S1:** Principal Component Analysis of RPKM (Reads Per Kilobase per Million reads) from the 6,745 genes common to the transcriptome and genome. Samples M^+^ (circle) and NM (triangles), in black, show the results for the dehiscent and indehiscent seed in the transcriptome. Samples M^+^ (circle) and NM (triangles), in white, show the corresponding results in the genome. Variation explained by each principal component is indicated on the axes.


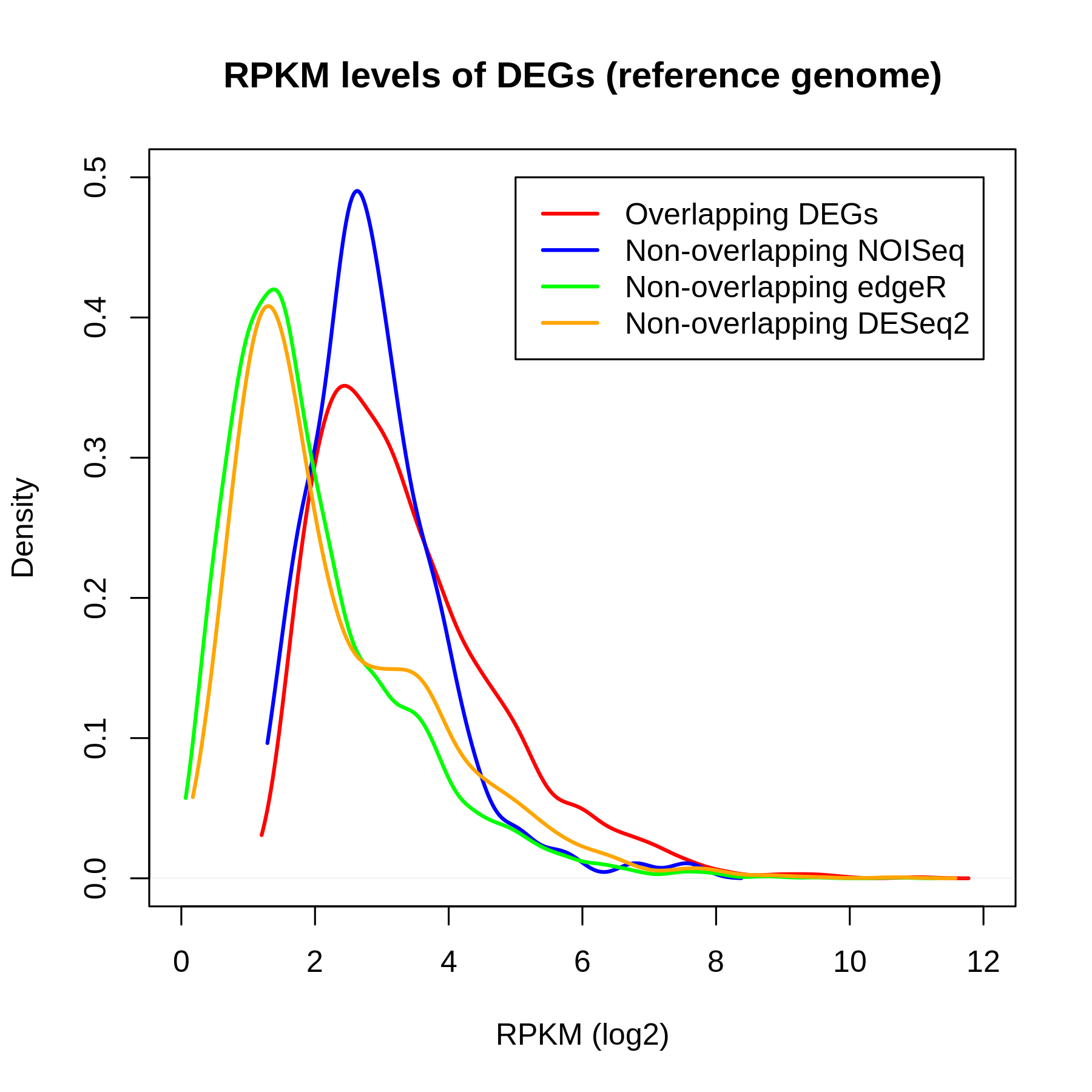


**Figure S2:** Density plot showing the RPKM values for the DEGs in the overlap between the three DEG calling packages (red), the DEGs called by NOISeq that are not in the overlap (blue), the DEGs called by edgeR that are not in the overlap (green), the DEGs called by DESeq2 that are not in the overlap (orange).


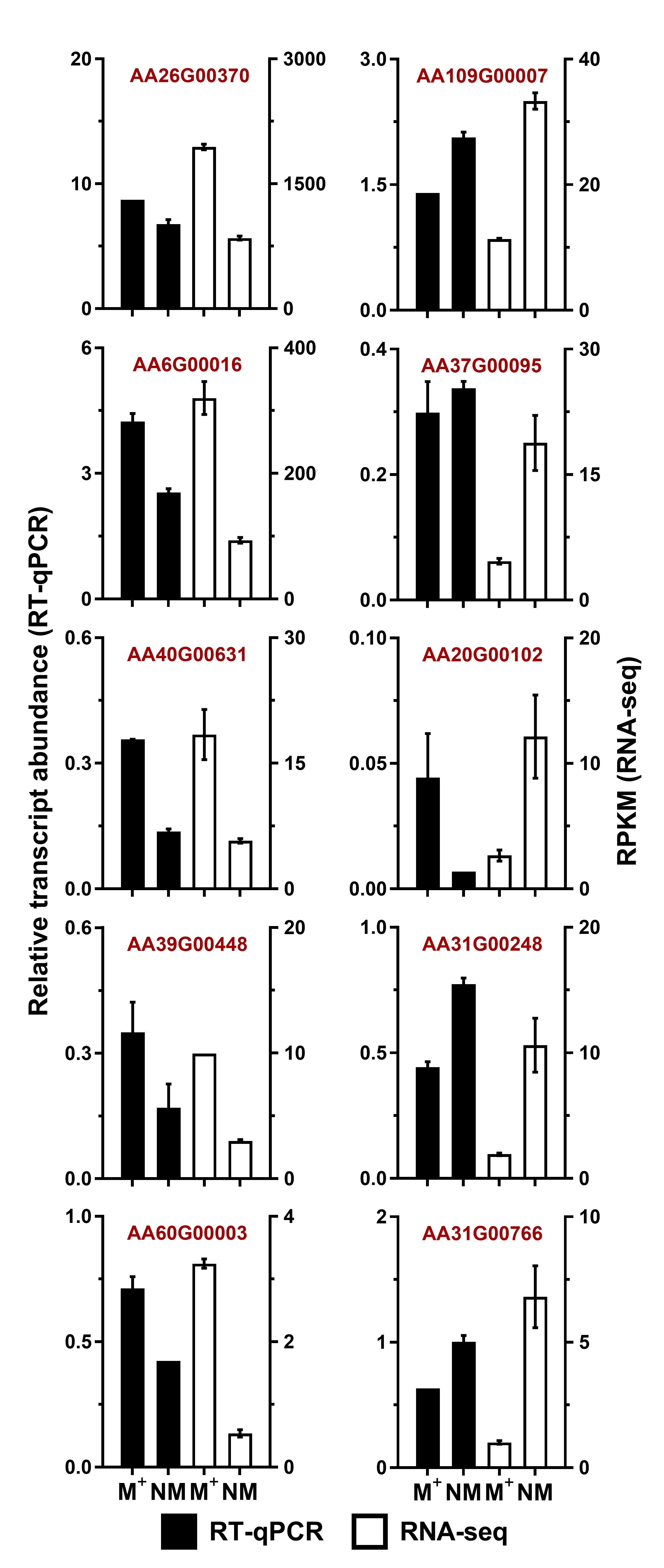


**Figure S3:** **qRT-PCR Expression.** qRT-PCR was used to measure the expression of ten candidate genes, identified as DEGs by RNA-seq, in a separate batch of seeds to the RNA-seq experiment (n = 5 per morphotype). Aethionema gene IDs are indicated. Error bars indicate ± 1 SEM.


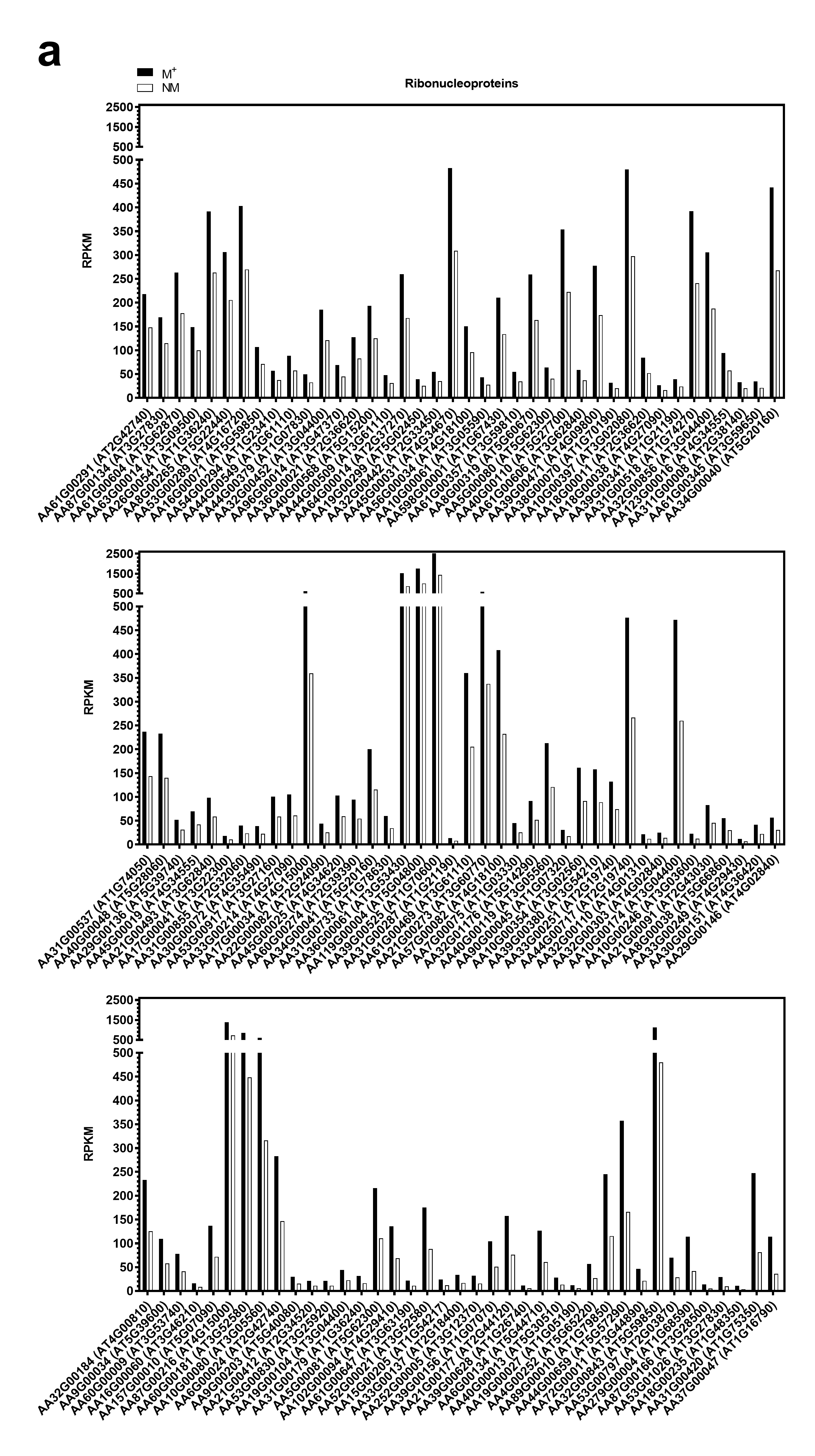

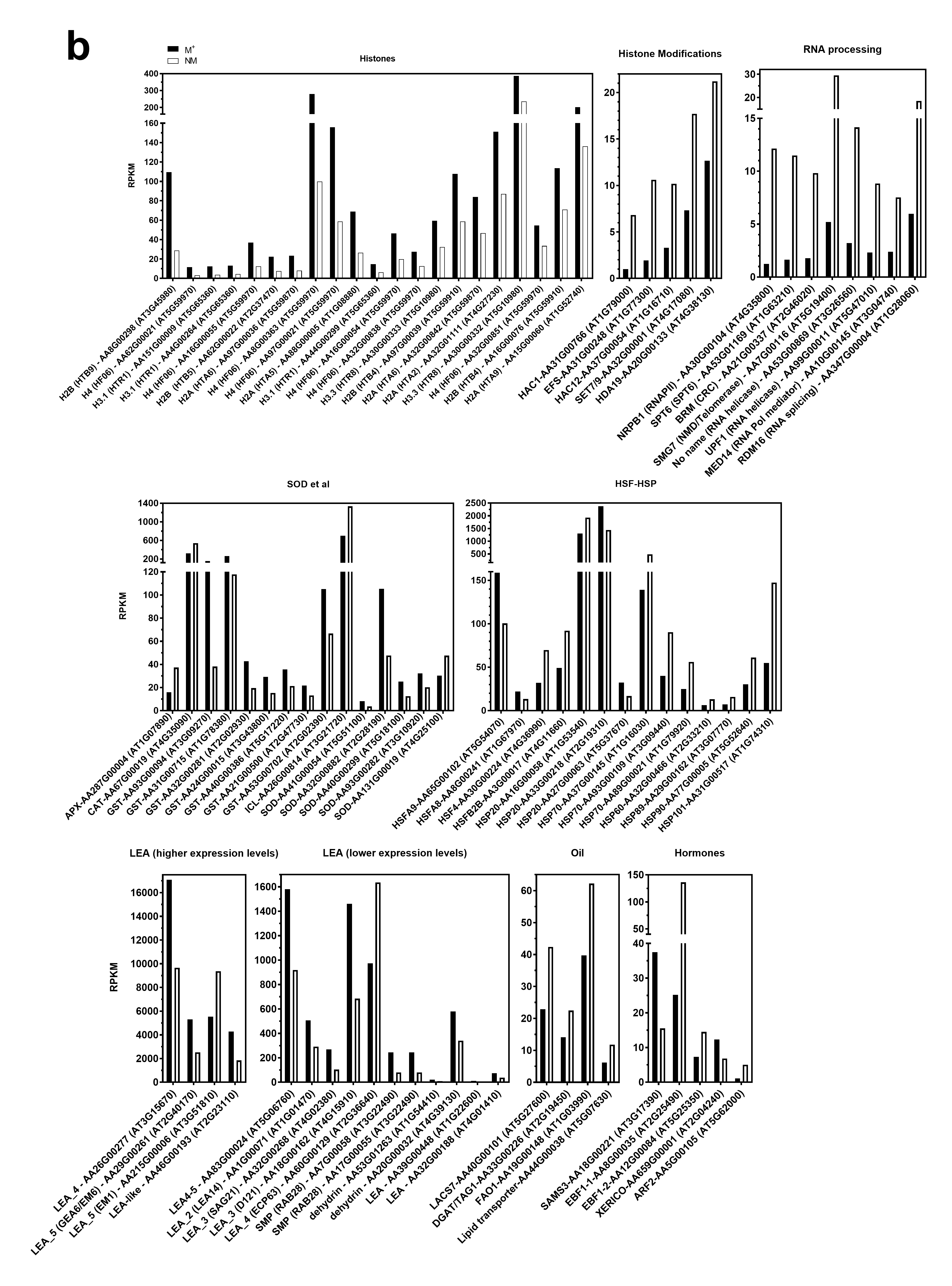


**Figure S4: Abundances of ribonucleoprotein transcripts (a) and transcripts from selected gene categories (b) from *Ae. arabicum* dimorphic seed DEG lists.** Gene names, Arabidopsis gene ID (ATx) and *Ae. arabicum* gene ID (AAx) indicated. M^+^ seeds (from DEH fruits) are compared to NM seeds (from IND fruits).

**Figure S5: Expression of hormone-related genes from published transcriptomes of developing and mature *A. thaliana* seeds [29-31, 74], whose orthologues were identified as *Ae. arabicum* dimorphic seed DEGs in this study.** Expression of *AtXERICO* (ABA – GA crosstalk), *AtEBF1* (Ethylene signaling), *AtSAMS3* (related to ethylene biosynthesis) and *AtARF2* (auxin signaling/ ethylene crosstalk) changes during Arabidopsis seed maturation.

*XERICO*, which is known to mediate between ABA and GA [A1] is up-reguated during *A. thaliana* seed maturation, and is among the NM-low DEGs. While *S*-adenosylmethionine (SAM) synthetase (SAMS3) which generates precursors for ethylene biosynthesis is down-regulated, the ethylene response EIN3-binding F-box protein (EBF1) is up-regulated during *A. thaliana* seed maturation (Figure S4, Additional file 6). This pattern is in agreement with dormancy induction in association with down-regulation of ethylene precursor production and down-regulation of ethylene signalling by EBF1 [A2]. Interestingly, transcripts of the putative *Ae. arabicum* orthologs are among the NM-low DEGs for SAMS3, and among the NM-high DEGs for EBF1. This is in agreement with the finding that *Ae. arabicum* NM seeds are deeper dormant compared to M^+^ seed [3]. Differences in ethylene-related gene expression in relation to the depth of dormancy are also evident among heterogenic seeds of *B. rapa* [A3]. Finally, mutants for the *A. thaliana* AUXIN RESPONSE FACTOR 2 (ARF2) are impaired in ethylene biosynthesis gene expression, silique development and have larger seeds [A4]. Interestingly, transcripts of the putative *Ae. arabicum* *ARF2* ortholog are among the NM-high DEGs (Figure S4b, Additional file 3). This suggests that the size difference between the dimorphic M^+^ and NM seeds of *Ae. arabicum* (Figure 1) could involve control by auxin-ethylene via ARF2.

A1. Piskurewicz U, Jikumaru Y, Kinoshita N, Nambara E, Kamiya Y, Lopez-Molina L: **The gibberellic acid signaling repressor RGL2 inhibits *Arabidopsis* seed germination by stimulating abscisic acid synthesis and ABI5 activity**. *The Plant Cell* 2008, **20**(10):2729-2745.

A2. Corbineau F, Xia Q, Bailly C, El-Maarouf-Bouteau H: **Ethylene, a key factor in the regulation of seed dormancy**. *Front Plant Sci* 2014, **5**:539.

A3. Puga-Hermida MI, Gallardo M, Rodriguez-Gacio MD, Matilla AJ: **The heterogeneity of turnip-tops (*Brassica rapa*) seeds inside the silique affects germination, the activity of the final step of the ethylene pathway, and abscisic acid and polyamine content**. *Functional Plant Biology* 2003, **30**(7):767-775.

A4. Okushima Y, Mitina I, Quach HL, Theologis A: **AUXIN RESPONSE FACTOR 2 (ARF2): a pleiotropic developmental regulator**. *Plant J* 2005, **43**(1):29-46.

**
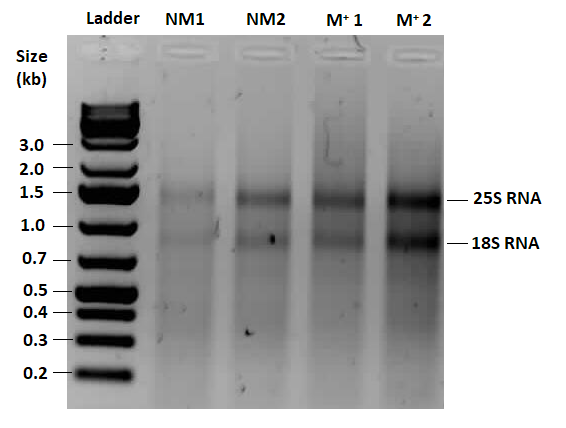
**

| **Sample Name:** | **Conc. (ng/µl)** | **A_260_/**  **A_280_** | **A_260_/**  **A_230_** | **Amount**  **(µg)** |
| --- | --- | --- | --- | --- |
| **M^+^1** | 389 | 2.03 | 1.99 | 18.8 |
| **M^+^2** | 293 | 2.00 | 2.34 | 14.2 |
| **NM1** | 153 | 2.12 | 1.88 | 7.34 |
| **NM2** | 204 | 2.10 | 2.08 | 9.77 |

**Figure S6: RNA integrity was checked by gel electrophoresis.** RNA was separated on an agarose gel. The presence of intact 25S and 18S RNA (indicated) was used to verify RNA integrity of NM and M^+^ RNA. Ladder is GeneRuler 1 kb Plus DNA Ladder (visible fragment sizes indicated), with 25S and 18S RNA bands indicated. RNA purity (A_260_ / A_280_, A_260_/A_230_) and concentration was determined using spectrophotometry.


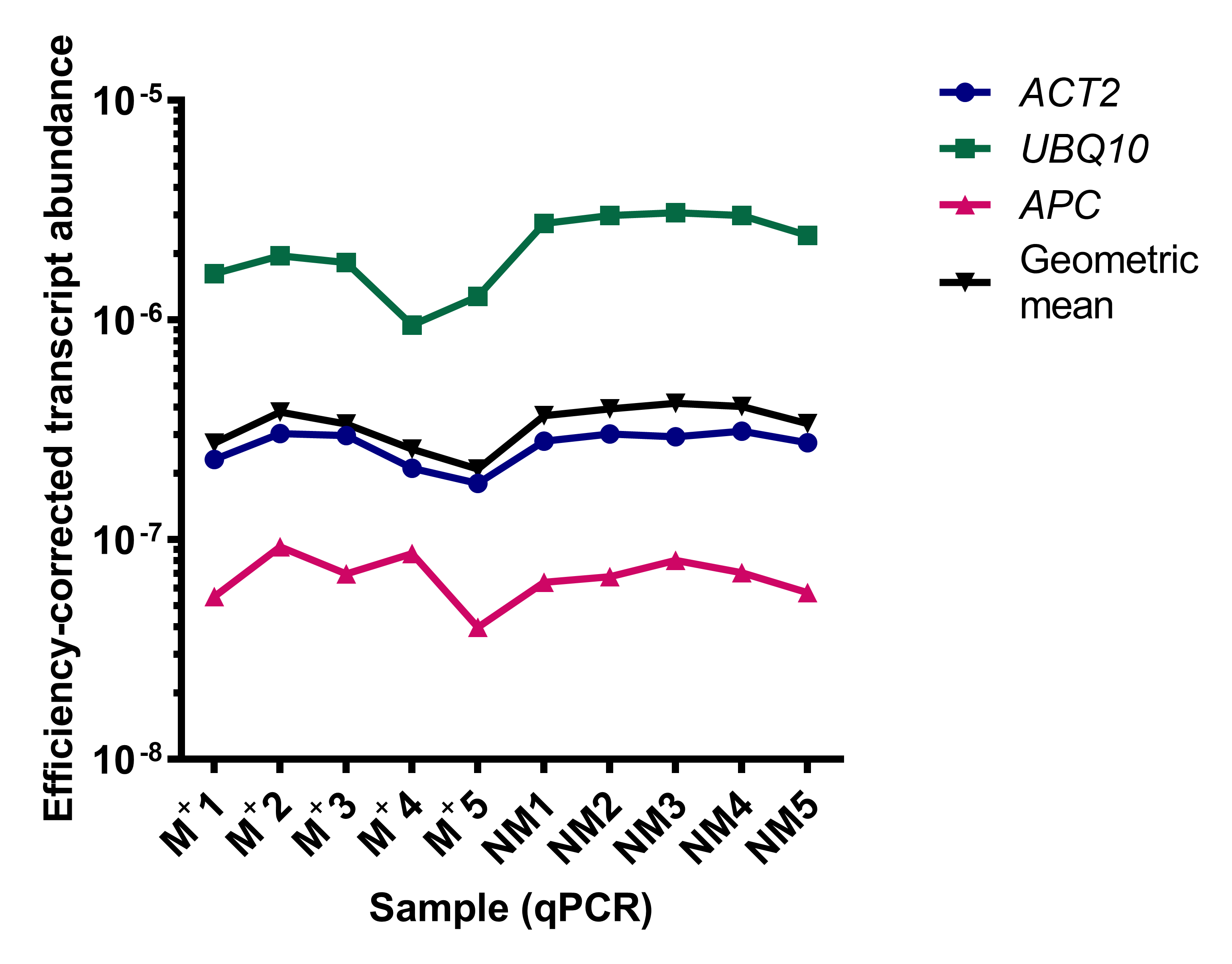


**Figure S7: Transcript abundance of candidate RT-qPCR normalisation reference genes dry in M^+^ and NM seed.** The efficiency corrected transcript abundance of *ACT2*, *UBQ10* and *APC* was calculated as (1+E_AveragePerAmplicon_)^−CT^_IndividualSample_, as previously described in Graeber *et al.,* 2011. The geometric mean of abundance of the genes is plotted for each sample, and was used to normalize RT-qPCR quantification of target genes.
